# Utilizing the Food–Pathogen Metabolome to Putatively Identify Biomarkers for the Detection of Shiga Toxin-Producing *E. coli* (STEC) from Spinach

**DOI:** 10.3390/metabo11020067

**Published:** 2021-01-25

**Authors:** Snehal R. Jadhav, Rohan M. Shah, Avinash V. Karpe, Robert S. Barlow, Kate E. McMillan, Michelle L. Colgrave, David J. Beale

**Affiliations:** 1 Consumer-Analytical-Safety-Sensory (CASS) Food Research Centre, School of Exercise and Nutrition Sciences, Deakin University, Burwood, VIC 3125, Australia; Snehal.Jadhav@deakin.edu.au; 2 Department of Chemistry and Biotechnology, Faculty of Science, Engineering and Technology, Swinburne University of Technology, Hawthorn, VIC 3122, Australia; rshah@swin.edu.au; 3 Land and Water, Commonwealth Scientific and Industrial Research Organization, Ecoscience Precinct, Dutton Park, QLD 4102, Australia; Avinash.Karpe@csiro.au; 4 Agriculture and Food, Commonwealth Scientific and Industrial Research Organization, Coopers Plains, QLD 4108, Australia; Robert.Barlow@csiro.au (R.S.B.); Kate.McMillan@csiro.au (K.E.M.); 5 Agriculture and Food, Commonwealth Scientific and Industrial Research Organization, St Lucia, QLD 4067, Australia; Michelle.Colgrave@csiro.au

**Keywords:** leafy greens, spinach, metabolomics, metabolic profiling, food pathogens, biomarker discovery

## Abstract

Shiga toxigenic *E. coli* (STEC) are an important cause of foodborne disease globally with many outbreaks linked to the consumption of contaminated foods such as leafy greens. Existing methods for STEC detection and isolation are time-consuming. Rapid methods may assist in preventing contaminated products from reaching consumers. This proof-of-concept study aimed to determine if a metabolomics approach could be used to detect STEC contamination in spinach. Using untargeted metabolic profiling, the bacterial pellets and supernatants arising from bacterial and inoculated spinach enrichments were investigated for the presence of unique metabolites that enabled categorization of three *E. coli* risk groups. A total of 109 and 471 metabolite features were identified in bacterial and inoculated spinach enrichments, respectively. Supervised OPLS-DA analysis demonstrated clear discrimination between bacterial enrichments containing different risk groups. Further analysis of the spinach enrichments determined that pathogen risk groups 1 and 2 could be easily discriminated from the other groups, though some clustering of risk groups 1 and 2 was observed, likely representing their genomic similarity. Biomarker discovery identified metabolites that were significantly associated with risk groups and may be appropriate targets for potential biosensor development. This study has confirmed that metabolomics can be used to identify the presence of pathogenic *E. coli* likely to be implicated in human disease.

## 1. Introduction

The World Health Organization has identified foodborne diseases as a major concern for public health and the world economy [1]. An estimated 600 million people fall ill every year from consuming contaminated foods. Among the leading causes of disease are bacterial pathogens such as pathogenic *Escherichia coli*.

*E. coli* are Gram-negative bacteria that are found in a wide variety of habitats including the gastrointestinal tract of animals and humans. Most *E. coli* are considered important microbiota members; however, some strains are known to be pathogenic and can cause diarrheal or systemic diseases in the host. The diarrheagenic *E. coli* consists of five pathotypes: enteropathogenic *E. coli* (EPEC), Shiga toxigenic *E. coli* (STEC) which also constitute the enterohemorrhagic (EHEC) strains, enteroaggregative *E. coli* (EAEC), enterotoxigenic *E. coli* (ETEC), and enteroinvasive *E. coli* (EIEC) [2].

Over the years, STEC strains have been associated with many incidences of foodborne diseases [3]. While in most people STEC infection results in mild, watery diarrhea, it can cause bloody diarrhea, and in vulnerable populations (such as the elderly, young, and immunosuppressed), can lead to more serious consequences such as hemolytic uremic syndrome (HUS), which can cause kidney failure [4]. STEC infections first rose to prominence in 1982 and again in 1993 with foodborne disease resulting from the ingestion of beef burgers contaminated with the strain *E. coli* O157:H7. Since then, more than 470 STEC strains have been isolated from humans but not all of them are pathogenic. Since 2011, O157 and six other serogroups O26, O45, O103, O111, O121, and O145 (also referred to as the “big 6”) have gained regulatory significance by the U.S. Department of Agriculture’s (USDA) Food Safety and Inspection Service (FSIS) [3].

Although contaminated meat has been frequently linked to STEC outbreaks, leafy greens, vegetables, and dairy products have also been linked to similar outbreaks [4]. According to the Centre for Disease Control (CDC), between 1973 and 2012, 46% of the total leafy vegetable outbreaks were caused by STEC strains [5]. In March 2020, the U.S. Food and Drug Administration (USFDA) released the 2020 Leafy Greens STEC Action Plan to reduce the number of STEC associated infections linked with leafy greens [6]. As the infectious dose of STEC is very low (between 10–100 CFU) and because there is a higher chance of consuming fresh produce in the raw state, it is very important to get a rapid and timely detection for such pathogens to ensure consumer confidence and safety [7].

According to the current USFDA’s Bacteriological Analytical Manual (BAM), the detection of STEC’s from leafy greens involves enriching the produce in an enrichment broth for about 24 h followed by screening for virulence genes and other markers using molecular techniques such as real-time PCR [5]. As opposed to other pathogens, the mere detection of *E. coli* is not enough. The characterization of the strain and its differentiation from other pathogenic and non-pathogenic *E. coli* are required. This is often a time-consuming process. The most recent advancement for the detection and characterization of pathogens has been whole-genome sequencing; however, in its current form, it also faces problems around the requirement of sophisticated bioinformatics, specialized laboratory equipment, data handling, and data ownership issues [2]. 

Metabolomics offers an approach from which rapid methods may be developed for screening potential food pathogens in complex food matrices via the discovery of novel biomarkers. In the last decade, this approach has shown promising progress in food traceability, composition, and safety [8]. Limited studies have explored the use of metabolomics to facilitate the rapid detection of pathogens [9,10,11,12,13]. It should be noted that most of these studies focused on pathogen detection at the species level from protein-rich matrices such as dairy and meat. The current study aims to investigate the application of metabolomics to detect STEC strains from fresh produce with “spinach” used as the model food. First, the metabolite profile of various STEC and non-STEC strains cultured in non-selective enrichment media (buffered peptone water (BPW)) was undertaken, investigating the metabolic differences amongst each strain in the supernatant and harvested pellet. This was expanded to incorporate artificially inoculated, commercially packaged (bagged) spinach with a cocktail of STEC and non-STEC to investigate the approach applied to a complex food matrix. The metabolite profile from the spiked spinach was then analyzed and compared with a suitable control (uninoculated) spinach sample using an untargeted metabolomics approach via gas chromatography coupled with mass spectrometry. This proof-of-concept study aims to determine if a metabolomics approach can be used to detect STEC contamination in fresh produce. Post validation, the potential biomarkers identified from this study can enable the development of a novel and rapid metabolomics-based diagnostic assay for detecting STECs from complex food matrices such as fresh produce.

## 2. Results and Discussion

Globally, the consumption of fresh produce has increased over the years with a change in dietary habits and lifestyle choices. Concurrently, the number of foodborne outbreaks associated with fresh produce has also increased, with STEC being major contributors [4,7,14]. The current study aimed to use an untargeted metabolomics approach to identify potential biomarkers specific to STEC contamination of fresh produce. 

**Figure 1:**
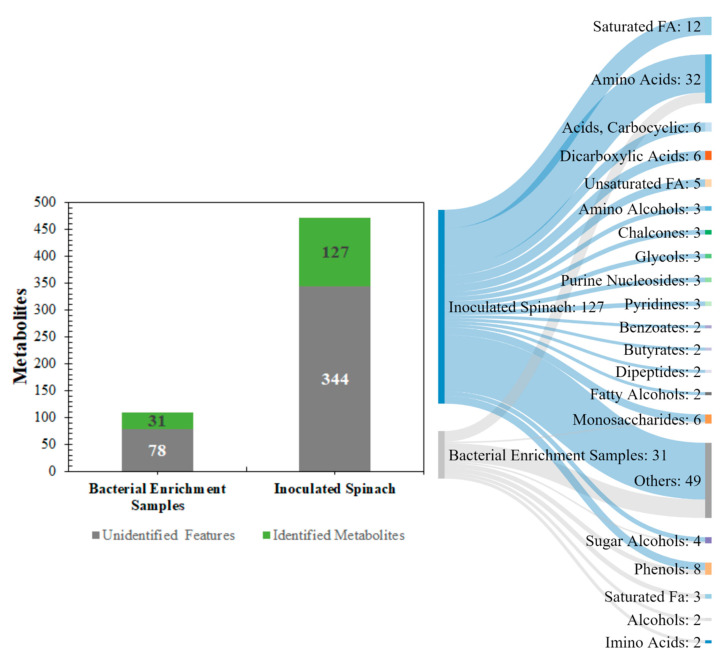
A global overview of the metabolic profiling outputs from the pathogenic *E. coli* experiments in buffered peptone water (BPW) cultures (*n* = 36) and inoculated spinach experiments (*n* = 58). The number of identified metabolites has been highlighted in green and the number of unidentified metabolites has been highlighted in grey.

Here, the supernatant and pellet samples from the bacterial and spinach enrichments were investigated for the presence of unique metabolites. As illustrated in Figure 1, a total of 109 metabolite features were detected across the bacterial enrichment samples, of which 31 were identified based on mass spectra fragmentation features and retention times. For the inoculated spinach, a total of 471 metabolite features were detected, out of which 127 were identified. The major metabolite classes identified across all samples, based on ChemRICH-class classification enrichment were amino acids, saturated fatty acids (FA), carboxylic acids, sugars, and sugar alcohols. 

### 2.1. Bacterial Enrichments

**Figure 2:**
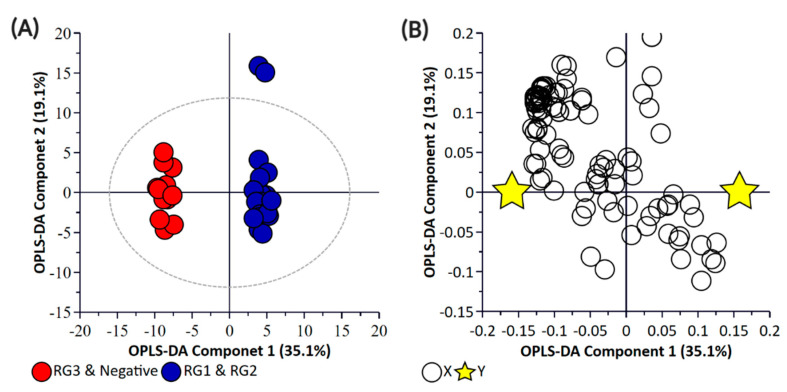
Orthogonal Partial Least Square-Discriminant Analysis (**A**) scatter plot and (**B**) loading plot of bacterial pellet samples collected from BPW cultures (*n* = 36; note, the negative group includes Salmonella). R^2^X (cum) = 0.698, R^2^Y (cum) = 0.989, Q^2^ = 0.879. Note, the ellipse presented in Figure 2A represents Hotelling’s T^2^ confidence limit (95%). Note: The colored circles in panel “**A**” represent each analyzed sample, while the yellow-colored stars in panel “**B**” indicate the average group position for each sample cluster, with the white circles representing the distribution of metabolite features between these groups.

**Figure 3:**
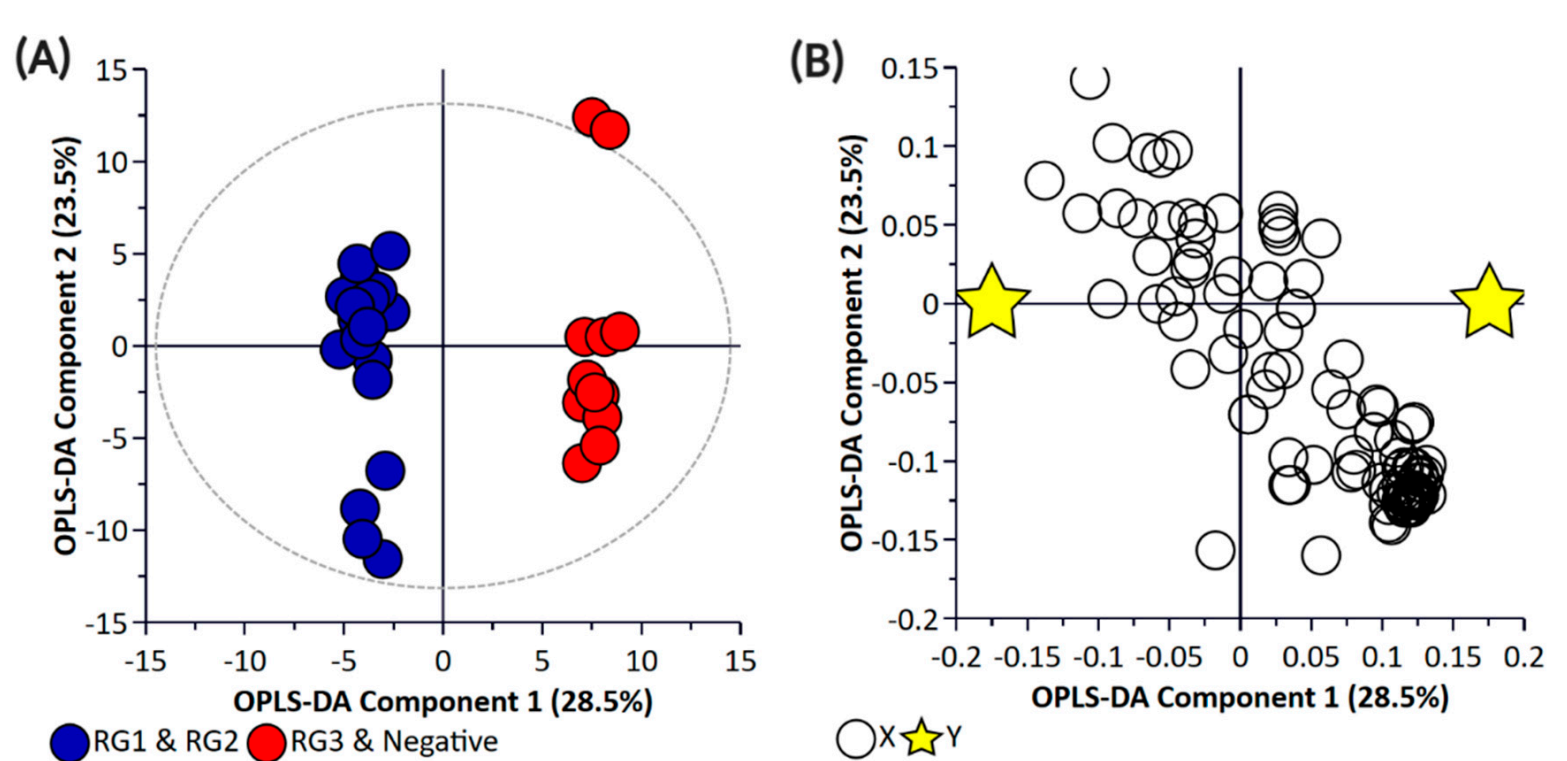
Orthogonal Partial Least Square-Discriminant Analysis (**A**) scatter plot and (**B**) loading plot of bacterial supernatant samples collected from BPW cultures (*n* = 36; note, the negative group includes Salmonella). R^2^X (cum) = 0.744, R^2^Y (cum) = 0.987, Q^2^ = 0.751. Note, the ellipse presented in Figure 3A represents Hotelling’s T^2^ confidence limit (95%). Note: The colored circles in panel “**A**” represent each analyzed sample, while the yellow-colored stars in panel “**B**” indicate the average group position for each sample cluster, with the white circles representing the distribution of metabolite features between these groups.

**Figure 4:**
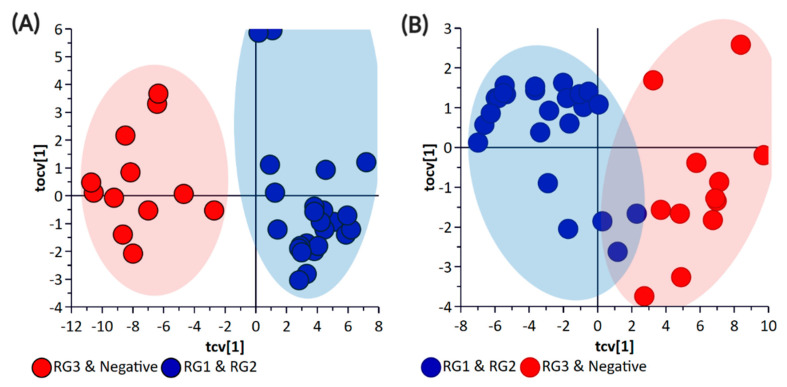
OPLS-DA Cross-Validation Scores plots for (**A**) bacterial pellet and (**B**) bacterial supernatant samples collected from BPW cultures (*n* = 36; note, the negative group includes Salmonella). The *F*-test statistic and *p*-Value based on a CV-ANOVA were 18.12 and <0.0001 for the pellet samples, and 5.78 and <0.0002 for the supernatant samples, respectively. Note: The colored circles in each panel represent each analyzed sample, and any overlap of the deviation of the samples from the origin (0,0) indicates the potential of the model to misclassify sample groupings.

Principal component analysis (PCA) and partial least square-discriminant analysis (PLS-DA) of the bacterial enrichments comprising the three risk groups (RG1, RG2, and RG3) with the “Negative” group did not show clear discrimination between the groups for the pellet (Supplementary Materials Figure S1) or the supernatant (Supplementary Materials Figure S2) samples. One of the reasons for this could be the higher metabolomic similarity between the different *E. coli* isolates. Therefore, a supervised orthogonal PLS-DA (OPLS-DA) analysis was performed. The pellet samples (Supplementary Materials Figure S3) and supernatant (Supplementary Materials Figure S4) samples showed clear discrimination between the different risk groups. As anticipated, RG1 isolates which include serogroups of regulatory significance were found to be more closely clustered with RG2 isolates. Isolates in both groups typically harbor *eae* and *stx* or possess additional genetic markers (e.g., pathogenicity islands or *stx-*associated O-antigen SNPs) consistent with isolates most likely to cause human disease. While RG3 samples could be separated from negative samples when bacterial pellets were analyzed, the same differentiation was not observed when the supernatants were analyzed. The lack of separation between RG3 and the negative groups likely confirms the absence of additional genetic markers in these samples and most likely reflects shared core biochemistry. As there is always a risk with overfitting data in supervised models such as OPLS-DA, and the percentage variation explained in the models being coupled with a predictability quotient (Q^2^), cross-validation of the OPLS models was undertaken (Supplementary Materials Tables S1 and S2, Figures S5 and S6). While the bacterial pellet model was found to be significant (*p*-value of 0.008), the data points were found to deviate from the axis origin which is indicative of a model with a high misclassification potential. As such, an additional model was generated that grouped RG1 and RG2 (as being of regulatory importance and similar virulence grouping) against the combined negative and RG3 groups of the pellet (Figure 2) and supernatant samples (Figure 3). This grouping resulted in the generation of a significantly improved model that was cross-validated (Figure 4) and were both found to be significant. 

**Figure 5:**
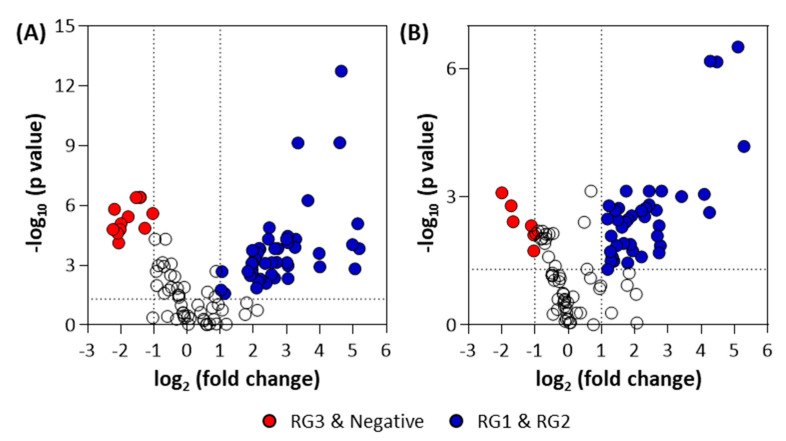
Volcano plots for (**A**) bacterial pellet and (**B**) bacterial supernatant samples collected from BPW cultures (*n* = 36; note, the negative group includes *Salmonella).* Note: The colored circles in each panel represent each detected significant metabolite. The open black circles represent statistical non-significant (*p*-value > 0.05) metabolites.

A volcano plot was generated of these groupings to identify the metabolites that were significantly altered for the pellet (Figure 5A) and supernatant samples (Figure 5B). A detailed summary of significant metabolites is provided in the Supplementary Materials (Supplementary Materials Tables S3 and S4). The statistically significant metabolites (*p* ≤ 0.05 and fold-change (FC) ≥ 2 or ≤0.5) that increased in the combined RG1 and RG2 pellet samples (as compared to the combined RG3 and negative) were 2-amino-2-methyl-1,3-propanediol, D-sphingosine, behenic acid, 2,3-dihydroxybiphenyl, acetohydroxamic acid, 3-hydroxyanthranilic acid, pelargonic acid, 4-aminophenol, DL-2-amino-3-phosphonopropionic acid, glycolic acid, halostachine, lauric acid, 2,6-dihydroxy-4-methoxytoluene, and 1-hexadecanol. The metabolites that decreased in pellet samples were pipecolic acid, trimethyllysine, L-methionine, cytidine, and N-acetyl-ornithine. On the other hand, the metabolites that significantly increased in the RG1 and RG2 supernatant samples were 2-amino-2-methyl-1,3-propanediol, 2,3-dihydroxybiphenyl, behenic acid, 2,3-butanediol, pelargonic acid, 4-aminophenol, acetohydroxamic acid, glycolic acid, 3-hydroxyanthranilic acid, halostachine, DL-2-amino-3-phosphonopropionic acid, and D-sphingosine. Epsilon-caprolactam and N-acetyl-ornithine were found to decrease in the supernatant samples. Further ANOVA analysis was done to compare various risk groupings (Tables S5 and S6, Supplementary Materials).

### 2.2. Spinach Enrichments

**Figure 6:**
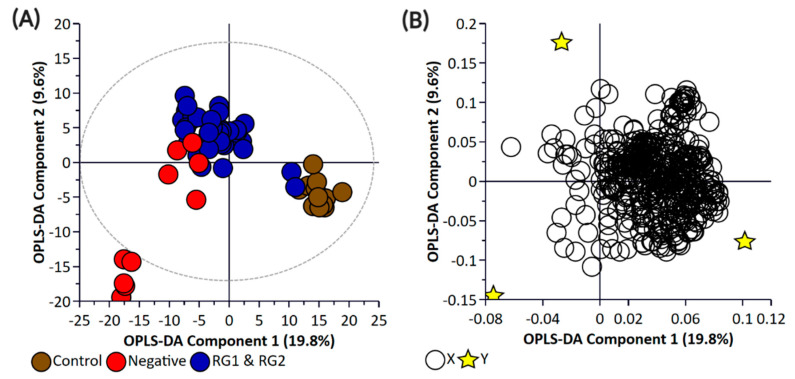
Orthogonal Partial Least Square-Discriminant Analysis (**A**) scatter plot and (**B**) loading plot of bacterial pellet samples collected from inoculated spinach samples (*n* = 58). R^2^X (cum) = 0.597, R^2^Y (cum) = 0.697, Q^2^ = 0.503. Note, the ellipse presented in Figure 6A represents Hotelling’s T^2^ confidence limit (95%). Note: The colored circles in panel “**A**” represent each analyzed sample, while the purple-colored stars in panel “**B**” indicate the average group position for each sample cluster, with the white circles representing the distribution of metabolite features between these groups.

**Figure 7:**
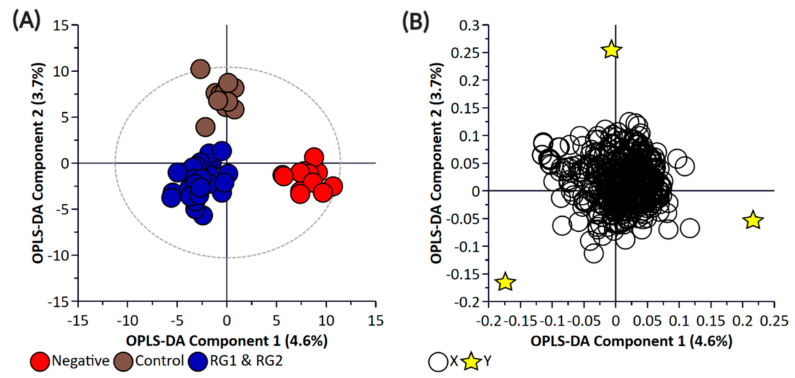
Orthogonal Partial Least Square-Discriminant Analysis (**A**) scatter plot and (**B**) loading plot of bacterial supernatant samples collected from inoculated spinach samples (*n* = 58). R^2^X (cum) = 0.512, R^2^Y (cum) = 0.882, Q^2^ = 0.481. Note, the ellipse presented in Figure 7A represents Hotelling’s confidence limit (95%). Note: The colored circles in panel “**A**” represent each analyzed sample, while the yellow-colored stars in panel “**B**” indicate the average group position for each sample cluster, with the white circles representing the distribution of metabolite features between these groups.

In the spinach experiments, attempts were made to differentiate samples spiked with RG1 or RG2 isolates from samples spiked with isolates from the negative group (which contained *Salmonella*) or uninoculated spinach (control group). Like the bacterial enrichment samples, both the pellet and the supernatant samples were used for performing the untargeted metabolomic profiling. PCA and PLS-DA analysis of the two risk groups (RG1, RG2) with the Negative and the Control groups did not show clear discrimination between the groups for the pellet (Supplementary Materials Figure S7) or the supernatant (Supplementary Materials Figure S8) samples. Therefore, a supervised OPLS-DA analysis was performed. Figures S9 and S10 (Supplementary Materials) represent the OPLS-DA plots for pellet and supernatant samples, respectively. The control samples (spinach only) were clearly separated from the spiked samples in both the pellet (R^2^X = 0.722, R^2^Y = 0.914, Q^2^ = 0.417) and supernatant samples (R^2^X = 0.635, R^2^Y = 0.945, Q^2^ = 0.429). More importantly, samples spiked with RG1 or RG2 isolates could be differentiated from both the negative and the control groups; however, the ability to distinguish between RG1- and RG2-spiked samples was more problematic with only marginal separation which was more pronounced in supernatant than pellet samples. However, like the bacterial OPLS-DA plots, cross-validation of these models indicated a high degree of misclassification potential (Supplementary Materials Tables S7 and S8, Figures S11 and S12). As such, an additional model was generated that grouped RG1 and RG2 (as being of regulatory importance and similar virulence grouping) against the negative and control groups of the pellet (Figure 6) and supernatant samples (Figure 7).

**Figure 8:**
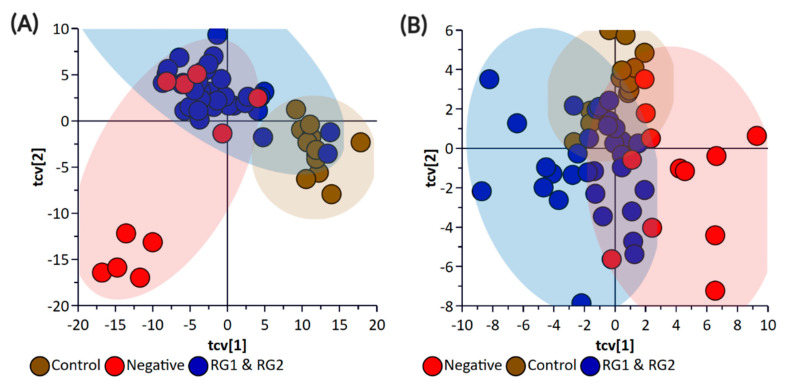
OPLS-DA Cross-Validation Scores plots for (**A**) bacterial pellet and (**B**) bacterial supernatant samples collected from inoculated spinach (*n* = 58; note, the negative group includes *Salmonella*). The F-test statistic and *p*-value based on a CV-ANOVA were 7.13 and <0.0001 for the pellet samples, and 3.9 and <0.0001 for the supernatant samples, respectively. Note: The colored circles in each panel represent each analyzed sample, and any overlap of the deviation of the samples from the origin (0,0) indicates the potential of the model to misclassify sample groupings.

Figure 8 illustrates the cross-validated score plots of these OPLS-DA models. As the focus herein was to putatively identify biomarkers that can distinguish these RG pathogens from the negative group and the control, this seemed appropriate. As illustrated in Figure 8, some of the negative group samples were misclassified as belonging to RG1 and RG2. Note that these samples were the negative *E. coli* cohort.

The following sections provide some deeper analyses of the differentially expressed metabolites relating to the various RG analyzed from inoculated spinach. Moreover, as the key focus here is to explore putatively identified biomarkers for the identification of RG1 and RG2 pathogens in spinach, a biomarker analysis was completed. 

### 2.3. Interaction between Spinach and Pathogenic E. coli Metabolomes for Pathway Mapping

**Figure 9:**
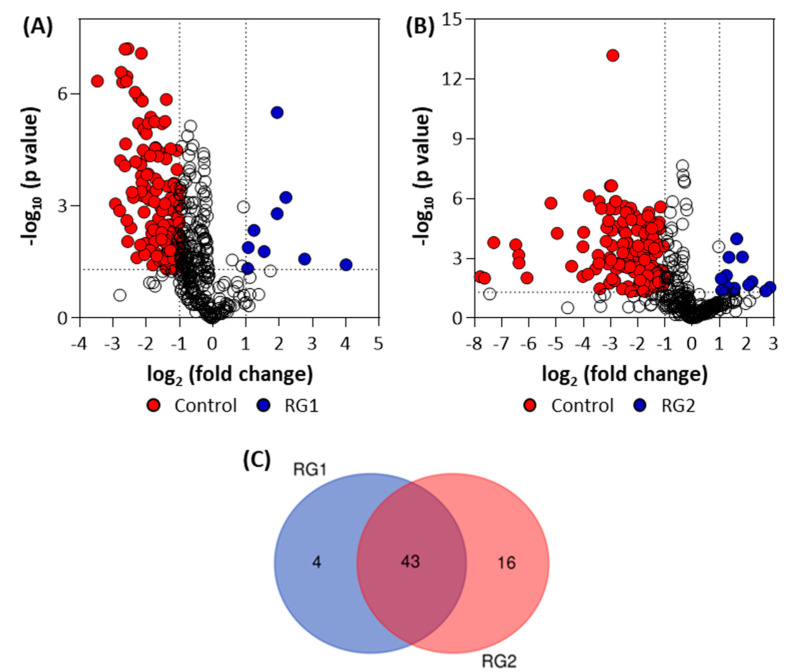
Volcano plots for bacterial pellet samples collected from (**A**) RG1-inoculated and (**B**) RG2-inoculated spinach samples (note, the negative group includes *Salmonella*) and (**C**) Venn diagram showing the statistically significant and identified “unique” metabolites. Note: The colored circles in panels A and B represent each detected significant metabolite. The open black circles represent non-significant (*p*-value > 0.05) metabolites.

Volcano plots (Figure 9) were generated to identify the statistically significant (*p* ≤ 0.05 and FC ≥ 2 or ≤0.5) metabolites from RG1 and RG2 pellet samples. From the identified metabolites in the spinach enrichments, a Venn diagram was constructed to identify the unique metabolites between the two groups (Figure 9).

**Figure 10:**
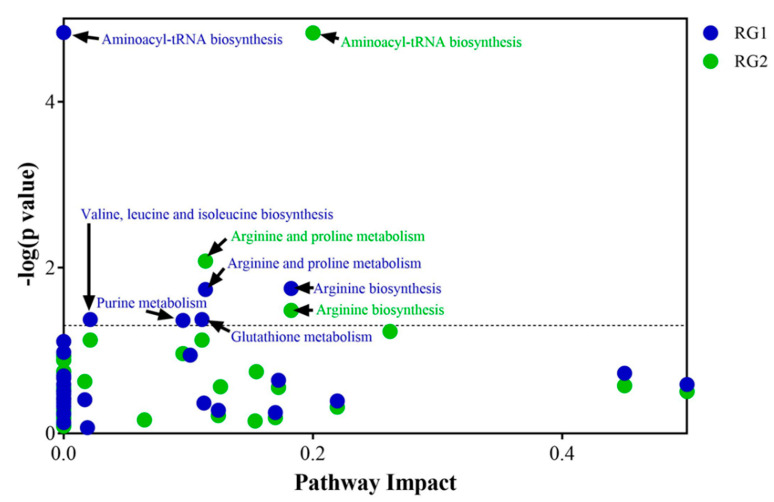
Pathway impact analysis of inoculated spinach. Statistically significant (*p*-value ≤ 0.05) pathways that were impacted by RG1 strains (blue dot points) and pathways impacted by RG2 strains (green dot points) have been indicated with black arrows.

To identify the metabolic pathways that are most likely induced during enrichment, pathway mapping analysis was performed using these significant metabolites. The 47 metabolites from RG1 (Supplementary Materials Table S9) and 59 metabolites from RG2 (Supplementary Materials Table S10) were then used to perform a pathway impact analysis in MetaboAnalyst (version 4.0) (Xia Lab, McGill University, Montréal, QC, Canada). Figure 10 indicates the statistically significant pathways (*p* ≤ 0.05) that were impacted in RG1 and RG2 strains growing in spinach enrichments. Amino acid tRNA biosynthesis, arginine biosynthesis, and arginine and proline metabolism were significantly affected in both the risk groups, whereas valine, isoleucine, and leucine biosynthesis; glutathione metabolism; and purine metabolism were mainly impacted in the RG1 group. The figure indicates that in both risk groups amino acid metabolism was most affected.

**Figure 11:**
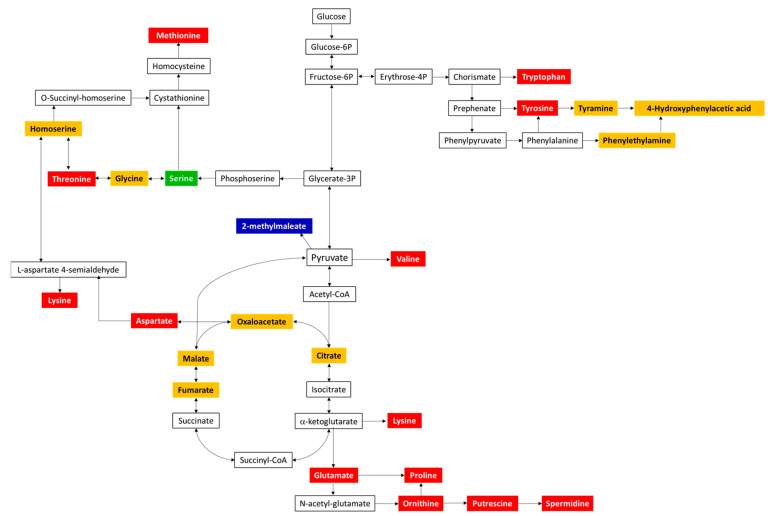
Pathway mapping of statistically significant ((*p* ≤ 0.05 and FC ≥ 2 or ≤0.5) metabolites of RG1 and RG2 identified in the inoculated spinach experiments. Significant metabolites common to both risk groups are highlighted in red, those belonging to RG1 only are highlighted in blue, and those belonging to RG2 only are highlighted in green. All other identified metabolites (but non-significant) are highlighted in orange. Unidentified metabolites are in black. L-methionine is the only amino acid that was upregulated; all other amino acids were downregulated.

To get a more holistic understanding of the amino acid pathways impacted, the identified metabolites (from significantly impacted metabolic pathways) from the pellet samples were mapped using the KEGG Mapper tool. Figure 11 highlights the metabolites mapped onto the “amino acid metabolism” pathway of *E. coli.*

As observed in Figure 11, several common and some unique amino acids were identified in both the risk groups; in comparison with the spinach-only sample, a significant increase in fold change was observed for the amino acid methionine (RG1-FC = 2.4, RG2-FC = 3.1), whereas a significant decrease was observed for threonine (RG1-FC = 0.48, RG2-FC = 0.37), lysine (RG1-FC = 0.21, RG2-FC = 0.14), aspartate (RG1-FC = 0.26, RG2-FC = 0.14), glutamate (RG1-FC = 0.45, RG2-FC = 0.33), proline (RG1-FC = 0.34, RG2-FC = 0.18), ornithine (RG1-FC = 0.23, RG2-F = -0.12), spermidine (RG1-FC = 0.41, RG2-FC = 0.42), valine (RG1-FC = 0.23, RG2-FC = 0.35), tyrosine (RG1-FC = 0.16, RG2-FC = 0.13), tryptophan (RG1-FC = 0.09, RG2-FC = 0.06), serine (RG2-FC = 0.42), and 2-methylmaleate (RG1-FC = 0.24).

Previous studies by Cevallos-Cevallos et al. and Li and Xu [11,12] have also identified changing amino acid levels during the metabolomic analyses of pathogenic *E. coli* strains. For instance, similar to the current study, Cevallos-Cevallos et al. [11] also observed a low level of the amino acid serine in the *E. coli* containing samples when compared to the control samples. The same study did not detect a significant amount of serine in the *E. coli* O157:H7 containing samples which were consistent with the findings in the current study as RG1 samples which included the *E. coli* O157:H7 serovar did not show the presence of serine. In the study by Li and Xu [12], a short enrichment period (4–8 h) was used before performing a targeted metabolomics study of pathogenic and non-pathogenic *E. coli* samples. In this study, lower levels of the amino acids N-acetyl-DL-glutamic acid and N-acetyl putrescine was observed in the pathogenic *E. coli* containing samples. Interestingly, the current study which involved a longer enrichment period (18 h) also detected a lower fold change of glutamate which is a precursor of N-acetyl-DL-glutamic acid, and L-ornithine which via decarboxylation produces putrescine [15]. Putrescine is a precursor of spermidine which was also identified in both the risk groups. Putrescine along with other polyamines such as spermine and spermidine can be found naturally in various foods or can also be produced by bacteria belonging to the Enterobacteriaceae family such as *E. coli* [15]. Detection of L-ornithine or other polyamines such as putrescine/spermidine could serve as an early indication of microbial spoilage in foods.

### 2.4. Pathogenic E. coli Biomarker Analysis in Spinach 

**Figure 12:**
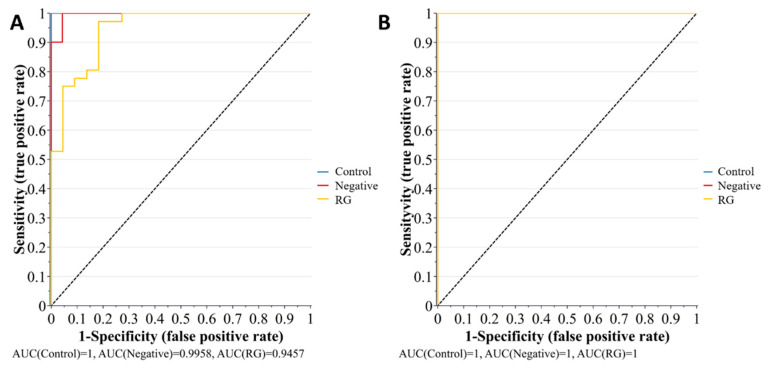
The plots represent multivariate ROC under the curve exploratory analysis of (**A**) pellet and (**B**) supernatant samples, for the metabolic profiling of bacterial risk groups (*n*(RG) = 32 and, *n*(Negative) = 12) inoculated into spinach enrichments. The outputs indicate the probability of biomarker predictability to the control (non-inoculated samples, *n* = 12), with a high sensitivity reflecting increased predictability of biomarkers. Note: for the biomarker analysis, the RG group comprises RG1 and RG2 combined.

The biomarker analysis was intentionally applied to the inoculated spinach, with specific focus given to the pelleted samples. This was done to account for the complexity of the spinach–pathogen–microbiome interaction and variation in the number of measured metabolites between the bacterial enrichment samples and the spinach samples. The biomarker analysis was performed using the SIMCA 16.1 Omix skin toolbox and the Biomarker analysis toolbox of MetaboAnalyst 4.0. The receiver operating characteristic (ROC) curve based on the area under the curve (AUC) was applied to the OPLS-DA dataset. A higher area under the curve (within a 95% confidence interval) is defined by the ratio between sensitivity (true positive rate) and specificity (false positive rate). A higher sensitivity/specificity ratio indicates greater model predictability. Figure 12 illustrates the multivariate ROC analysis for the pellet and supernatant samples obtained from the inoculated spinach experiments. 

However, the multivariate ROC analysis provided only the overall behavior of the groups. Therefore, to understand the contribution of individual metabolites as potential biomarkers, univariate ROC analysis was also performed using the “Biomarker analysis” toolbox of MetaboAnalyst 4.0. It was observed that the predictability of biomarkers was higher in the pellet (Q^2^(cum) = 52.1%) with respect to the supernatant (Cumulative Q^2^(cum) = 39.7%). 

Metabolites such as lysine (AUC = 0.93, Log_2_FC = −7.27), tyrosine (AUC = 0.95, Log_2_FC = −6.59), adenosine (AUC = 0.88, Log_2_FC = −4.8) cellotetraose (AUC = 0.96, Log_2_FC = −2.63), norleucine (AUC = 0.97, Log_2_FC = −3.82), and serine (AUC = 0.72, Log_2_FC = −1.04) showed depletion in the risk groups. Conversely, metabolites such as L-methionine (AUC = 0.79, Log_2_FC = 2.21) and 4-hydroxycinnamate (AUC = 0.68, Log_2_FC = 2.0) were observed to increase in pellet samples containing risk groups (RG) (Supplementary Materials Figure S13A).

Similarly, when compared to the negative controls, metabolites such as linoleate (AUC = 1, Log_2_FC = 5.81), 4-isopropylbenzoate (AUC = 0.99, Log_2_FC = 8.48), 3,4-dihydroxymandelate (AUC = 0.92, Log_2_FC = 5.09), and stearate (AUC = 0.94, Log_2_FC = 5.15) showed elevation in the RG pellet samples. On the other hand, tryptophan (AUC = 0.9, Log_2_FC = −3.36) and 3-aminoisobutyrate (AUC = 0.81, Log_2_FC = −1.62) were predominantly depleted metabolites in the RG pellet samples (Supplementary Materials Figure S13B). 

A few statistically significant metabolites (*p* ≤ 0.05) were identified in both RG1 and RG2, and therefore we compared the two groups to determine the differences in their output. Unlike the observations earlier (when compared to the control samples), this comparison yielded fewer metabolites with high AUC (>0.9). The major metabolites were 4-2-hydroxyethylphenol (AUC = 0.94, Log_2_FC = 2.08) 4-hydroxyphenylacetate (AUC = 0.78, Log_2_FC = 1.27), inosine (AUC = 0.75, Log_2_FC = 1.19), and serine (AUC = 0.72, Log_2_FC = 1.34) showing increased levels in RG 1 (Supplementary Materials Figure S13C).

4-Hydroxyphenylacetic acid is primarily a plant-based metabolite and is generated as the downstream product of phenylalanine and tyrosine metabolism. Some *E. coli* strains have the gene functions for translation of tyrosine aminotransferase, aspartate aminotransferase, histidinol-phosphate aminotransferase, and 4-hydroxyphenylacetate 3-monooxygenase enzymes, which facilitate this metabolism [16]. The depletion of tyrosine and tryptophan in the RG samples in our study indicated this activity. Inosine is one of the important intermediates of nucleotide metabolism. In a recent study [17], the effect of *E. coli* O157:H7 infection in *Caenorhabditis elegans* (nematode) indicated the role of increased inosine levels in pathways related to nucleotide salvaging and, to some extent, lipid oxidation. This increase was observed to alleviate the cellular damage in the nematode caused by *E. coli* O157:H7. The importance of inosine was also shown in a recent study which indicated inosine-containing alleles in the *E. coli* O157:H7 genes which code for heat-stable enterotoxin type I [18]. The increased levels of serine and methionine in *C. elegans* infected with *E. coli* O157:H7 has been indicative of increased toxicity, caused by upregulated methionine and homocysteine pathways [17,19]. Our observations align well with these studies. However, a proteomics-based approach will further establish the outputs of this study, and the metabolic behavior and virulence expressions of various *E. coli* strains.

## 3. Materials and Methods

### 3.1. Bacterial Strains and Culture Media

A total of 20 *E. coli* isolates from the CSIRO STEC culture collection, harboring various combinations of genes encoding Shiga toxin (*stx*) and intimin (*eae*) and belonging to a range of serogroups, were selected for inclusion in the study. The isolates were assigned to risk groupings 1 to 3 which were based on their regulatory importance or pathogenic potential. Risk Group 1 (RG1) contains STEC of regulatory importance known as Top7 STEC which includes O157 and the Big6 serogroups (O26, O45, O103, O111, O121, and O145). Risk Group 2 (RG2) contains non-Top7 STEC, potential enterohaemorrhagic *E. coli* (pEHEC) and atypical enteropathogenic *E. coli* (aEPEC), and risk group 3 (RG3) is comprised of *eae*-negative STEC. A fourth grouping, designated as “negative”, included five generic *E. coli* and five *Salmonella enterica* isolates. A summary of the isolate information is shown in Table 1. All isolates were recovered from freezer stocks (−80 °C) using tryptic soya agar (Oxoid, Basingstoke, UK) incubated overnight at 37 °C. The resulting cultures were sub-cultured to confirm purity and were subsequently tested for *stx* and *eae* by conventional multiplex PCR [20].

### 3.2. Sample Preparation 

#### 3.2.1. Bacterial Enrichments

Bacterial enrichments were prepared by first enriching each isolate (Table 1) in 10 mL of buffered peptone water (BPW; Oxoid, Basingstoke, UK) overnight at 37 °C. The resulting enrichments were then diluted 1 in 1000 using BPW and a 30 µL aliquot was subsequently used to inoculate 30 mL of BPW which was then incubated at 37 °C for 18 ± 2 h. A minimum of four replicates was prepared for each risk grouping with a maximum of five isolates included in any one enrichment. As risk grouping 1 comprised 10 isolates, enrichments were prepared such that they contained a maximum of two serogroups (e.g., O26 and O111). Sterile, uninoculated BPW was enriched and used as negative growth control.

#### 3.2.2. Spinach Enrichments

Spinach samples were acquired from three separate supermarkets located in South East Queensland, Australia. Spinach samples were prepared by combining 25 g of spinach with 250 mL of BPW. All spinach samples were stomached for 60 s at four strokes per second (Interscience, St Nom La Breteche, France) before the addition of a bacterial inoculum. Bacterial inoculums were prepared for each risk grouping using the following approach. Each isolate was initially enriched in BPW overnight at 37 °C before being diluted 1 in 1000 using BPW. A cocktail inoculum for each risk grouping was then prepared by combining equal volumes of the isolates and subsequently diluting it 1 in 10 in BPW. A 1 mL aliquot of the resulting cocktail was then added to each sample, as required, to obtain an overall inoculum of between 100 and 1000 CFU/g. Samples were incubated overnight at 37 °C for 18 ± 2 h before being processed further. Four replicates were prepared for all spinach/risk grouping combinations and uninoculated spinach samples were included as controls.

### 3.3. Metabolomic Analysis

#### 3.3.1. Preparation of Cell Pellet for Metabolomic Analysis

Following enrichment, a sample aliquot (1 mL) was transferred to a 10 mL centrifuge tube for quenching of metabolism. Quenching solution (4 mL) comprising of 60:40 (*v/v*) methanol:water containing ammonium hydrogen carbonate to a final concentration of 0.85% (*w/v*) was added to the aliquot. The cellular mass was pelleted in a centrifuge (Sigma 4K-15; Sigma, London, UK) for 10 min at 4800× *g* and −8 °C. The cell pellet was stored at −80 °C until further analysis.

#### 3.3.2. Preparation of Cell Media for Metabolomic Analysis

A small volume of sample (1 mL) was transferred to a microcentrifuge and subjected to centrifugation at 13,500× *g* for 5 min to remove any cell debris and suspended cells. The supernatant (1.5 mL) was then transferred into fresh microfuge tubes, lyophilized at a low temperature, and stored at −80 °C until further analysis. 

#### 3.3.3. Metabolite Extraction 

The lyophilized samples were reconstituted in 1 mL methanol consisting of 100 μL internal standard (IS1) solution (20 mg mL^−1^ each of glycine-d5 and L-alanine-d4 in methanol) was added to each labeled 2 mL centrifuge tubes. The mixture was thoroughly vortexed for 2 min followed by centrifugation at 573× *g* at 4 °C for 15 min. A 50 μL aliquot of the supernatant was then transferred into 2 mL vials and evaporated to dryness in a vacuum concentrator (CentriVap Concentrator, Kansas City, MO, USA) at 40 °C. Myristic acid-d27 was added (0.2 mg mL^−1^; 10 µg after drying) as a secondary internal standard (IS2) and, the samples were re-dried. 

#### 3.3.4. GC-MS Analysis

The dried extracts were derivatized “in time”, followed by a 1-h holding time, before injection into a GC-MS as per previously reported [21], with some modifications. Briefly, trimethylsilyl (TMS) derivatives were formed by adding 20 μL of methoxyamine hydrochloride (MOX, 20 mg mL^−1^ in pyridine) and 40 μL of N, O-bis(trimethylsilyl)trifluoroacetamide (BSTFA) containing 1% trimethylchlorosilane (TMCS) following a two-step derivatization protocol implemented in-time using a Gerstel MPS autosampler (Gerstel GmbH & Co. KG, Deutschland, Germany). The derivatized samples were then analyzed using an Agilent 6890B GC oven coupled with a 5973A MS detector (Agilent Technologies, Mulgrave, VIC, Australia). The GC-MS system was equipped with a 30 m DB-5MS column (0.25 mm ID, 0.25 µm film thickness). The splitless method was used with 1 µL volume; the oven was held at an initial temperature of 70 °C for 2 min before increasing to 325 °C at 7.5 °C min^−1^; the final temperature was held for 4.5 min. Data acquisition and spectral analysis were performed using MassHunter. Qualitative identification of the compounds was performed according to the Metabolomics Standard Initiative Chemical Analysis Workgroup using the Agilent Fiehn Metabolomics Library (G166766A, Agilent Technologies, Santa Clara, CA, USA). For peak integration, a 5-point detection filtering (default settings) was set with a start threshold of 0.2 and a stop threshold of 0.0 for 10 scans per sample. 

### 3.4. Data Analysis

The data were imported and log-transformed using SIMCA 16 (MKS Data Analytics Solutions, Uméa, Sweden). Partial Least Square-Discriminant Analysis (PLS-DA) was performed by finding successive orthogonal components from the two or more datasets with maximum squared covariance and was subsequently used to identify the common relationships among the multiple datasets. All supervised models were cross-validated using a default 7-fold cross-validation method and CV-ANOVA statistic as indicated previously [22].

MetaboAnalyst 4.0 [23] and KEGG Mapper [24] were used for metabolic pathway analysis [25], and metabolites with a Benjamini–Hochberg-adjusted *p*-value of ≤ 0.05 and Fold Changes (FC) of <0.5 (downward regulation) or >2.0 (upward regulation) were considered to be statistically significant. Biochemical pathway enrichment analysis was performed using ChemRICH (http://chemrich.fiehnlab.ucdavis.edu/), a novel statistical approach based on chemical similarity [26]. Enrichment *p*-values and FC were obtained using SIMCA. A Venn diagram was drawn using an online web tool (http://bioinformatics.psb.ugent.be/webtools/Venn/). Sankey diagrams were created using the SankeyMATIC online web tool (http://sankeymatic.com/), respectively.

## 4. Conclusions

STEC are an important cause of foodborne disease globally, with many outbreaks linked to the consumption of contaminated foods such as leafy greens and red meat. STEC is considered an adulterant in raw, non-intact beef products in the USA. Consideration is being given to microbiological surveys and enhanced sampling protocols for STEC in leafy greens; however, methods remain laborious and provide little opportunity for supply chains to assess and mitigate food safety risks, with the emphasis remaining on end-product testing. The use of untargeted metabolomics may yield alternative pathogen detection tools that overcome these limitations and lead to the development of in-line risk mitigation strategies. This proof-of-concept study has shown that the use of such an approach does enable STEC, of human and regulatory significance, to be differentiated from other STEC and Enterobacteriaceae. Furthermore, it enabled the identification of specific biomarkers for which rapid detection tools and biosensors can be subsequently developed that facilitate potentially cheaper and quicker detection systems that may be utilized in a biosensor-based risk mitigation approach to food production and processing and suggests that it could be extended to other pathogens/food combinations (i.e., red meat).

## Figures and Tables

**Table 1:** Category, serogroup, virulence profile, and risk groupings of isolates included in the study. Note: The column “Isolates” refer to ID of individual strains from CSIRO the CSIRO STEC culture collection.

Category	Isolates	Serogroups	Virulence Profiles	Risk Grouping (RG)
Top7 STEC	EC 1543, 2941, 2996a, 2997a, 4399a, 4400a, 4412a, 4419a, 4433a, and 5054a	O157, O26, O45, O103, O111, O121, and O145	*stx_1_, stx_2_* and *eae; stx_1_* and *eae; stx_2_* and *eae*	1
Non-Top7 STEC	EC 3633a, 3639a, and 3683a	O84, O177, and O182	*stx_1_, stx_2_* and *eae; stx_1_* and *eae*	2
pEHEC/aEPEC	EC 801, 1646a, 3989a, and 4560a	O26, O103 and O145	*Eae* only	2
*Eae*-negative STEC	EC 4742a, 4819c, and 4852b	Unknown	*stx_1_* and *stx_2_*; *stx_1_* only	3
Generic *E. coli*	Five cattle isolates	Unknown	NA	Negative
*Salmonella*	Five cattle isolates	Unknown	NA	Negative

## Data Availability

The data presented in this study are avaible on request from the corresponding author. The data are not publicaly available due to intellectual property restrictions.

## Supplementary Materials

The following are available online at https://www.mdpi.com/2218-1989/11/2/67/s1, Figure S1: Principal component analysis and partial least square-discriminant analysis of bacterial pellet samples collected from buffered peptone water cultures, Figure S2: Principal component analysis and partial least square-discriminant analysis of bacterial supernatant samples collected from buffered peptone water cultures, Figure S3: Orthogonal partial least square-discriminant analysis of bacterial pellet samples collected from buffered peptone water cultures, Figure S4: Orthogonal partial least square-discriminant analysis of bacterial supernatant samples collected from buffered peptone water cultures, Figure S5: Cross-validation scores plots of the OPLS-DA bacterial pellet model, Figure S6: Cross-Validation (CV) Scores plots of the OPLS-DA bacterial supernatant model, Figure S7: Principal component analysis and partial least square-discriminant analysis of bacterial pellet samples collected from inoculated spinach samples, Figure S8: Principal component analysis and partial least square-discriminant analysis of bacterial supernatant samples collected from inoculated spinach samples, Figure S9: Orthogonal partial least square-discriminant analysis of bacterial pellet samples collected from inoculated spinach samples, Figure S10: Orthogonal partial least square-discriminant analysis of bacterial supernatant samples collected from inoculated spinach samples, Figure S11: Cross-Validation (CV) Scores plots of the OPLS-DA bacterial supernatant model, Figure S12: Cross-Validation (CV) Scores plots of the OPLS-DA inoculated spinach supernatant model, Figure S13: The plots indicate the top 15 validated biomarkers, as analyzed by PLS-DA classification and feature ranking through a Monte-Carlo cross-validation method, Table S1: Cross-validation (CV)-ANOVA of the OPLS-DA bacterial pellet model (Figure S3), Table S2: Cross-validation (CV)-ANOVA of the OPLS-DA bacterial supernatant model (Figure S4), Table S3: Significant metabolites in bacterial pellet samples collected from buffered peptone water cultures, Table S4: Significant metabolites in bacterial supernatant samples collected from buffered peptone water cultures, Table S5: ANOVA analysis of bacterial pellet samples collected from buffered peptone water cultures, Table S6: ANOVA analysis of bacterial supernatant samples collected from buffered peptone water cultures, Table S7: Cross-validation (CV)-ANOVA of the OPLS-DA inoculated spinach pellet model, Table S8: Cross-validation (CV)-ANOVA of the OPLS-DA inoculated spinach supernatant model, Table S9: Significant metabolites identified in bacterial pellet from RG1-inoculated spinach samples, Table S10: Significant metabolites identified in bacterial pellet from RG2-inoculated spinach samples.
