# Supplementary material for: Utilizing the Food–Pathogen Metabolome to Putatively Identify Biomarkers for the Detection of Shiga Toxin-Producing E. coli (STEC) from Spinach
Source: Metabolites. 2021 Jan 25;11(2):67. doi: 10.3390/metabo11020067 (PMC7911566; doi:10.3390/metabo11020067)
Supplement: Supplementary file 1 [file metabolites-11-00067-s001.pdf]

Supplementary

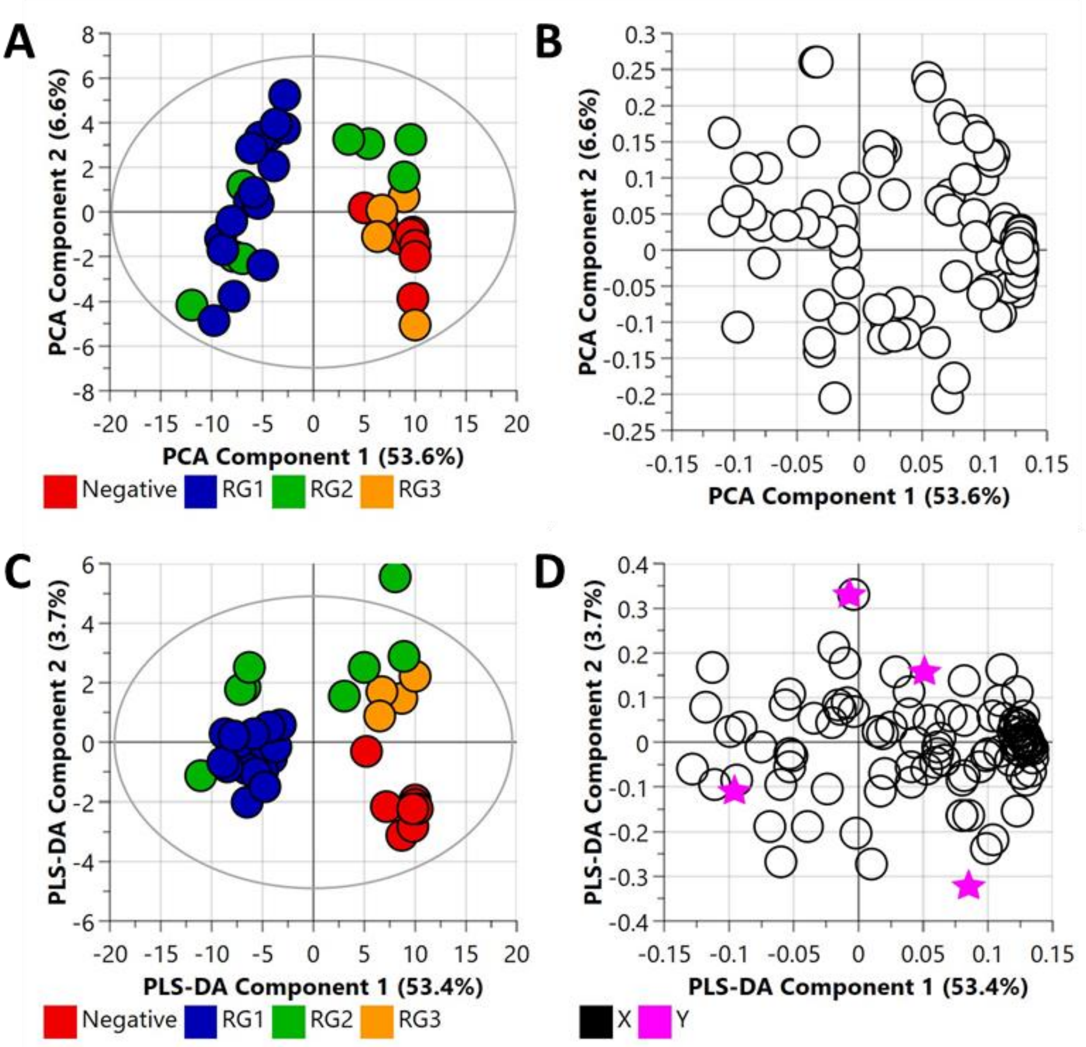

Figure S1. Principal component analysis (PCA) (R2X (cum) = 0.653, Q2 = 0.521) and partial least square-discriminant analysis (PLS-DA) (R2X (cum) = 0.571, R2Y (cum) = 0.494, Q2 = 0.312) of bacterial pellet samples collected from buffered peptone water (BPW) cultures. *Note, the eclipse presented in Figure S1A and S1C represents the Hotelling's confidence limit (95%). Note: The colored circles in panel "A" and "C" represent each analysed samples, while the purple-colored stars in panel "B" and "D" indicate the average group position for each sample cluster, with the white circles representing the distribution of metabolite features between these groups.*

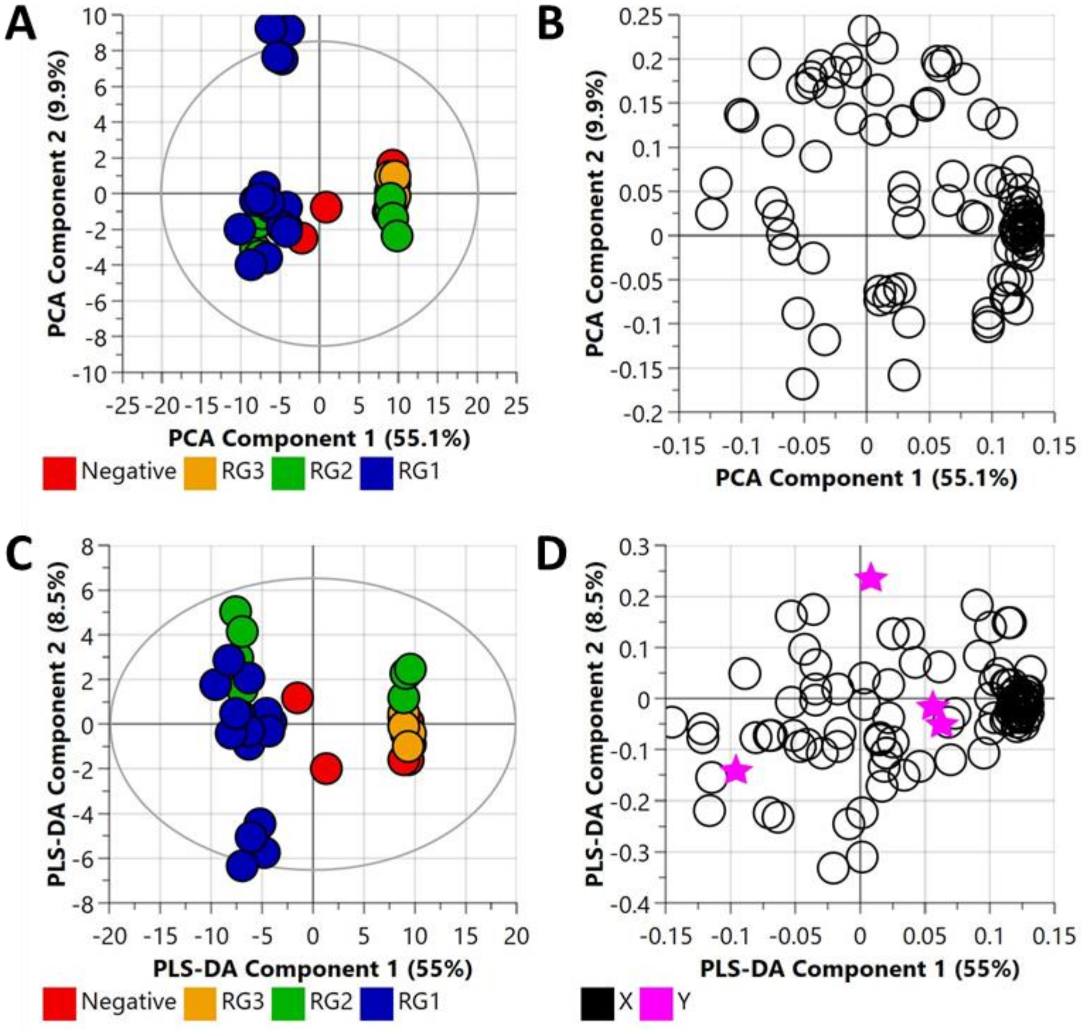

Figure S2. Principal component analysis (PCA) (R2X (cum) = 0.649, Q2 = 0.581) and partial least square-discriminant analysis (PLS-DA) (R2X (cum) = 0.696, R2Y (cum) = 0.697, Q2 = 0.347) of bacterial supernatant samples collected from buffered peptone water (BPW) cultures. *Note, the eclipse presented in Figure S2A and S2C represents the Hotelling's confidence limit (95%). The colored circles in panel "A" and "C" represent each analysed samples, while the purple-colored stars in panel "B" and "D" indicate the average group position for each sample cluster, with the white circles representing the distribution of metabolite features between these groups.*

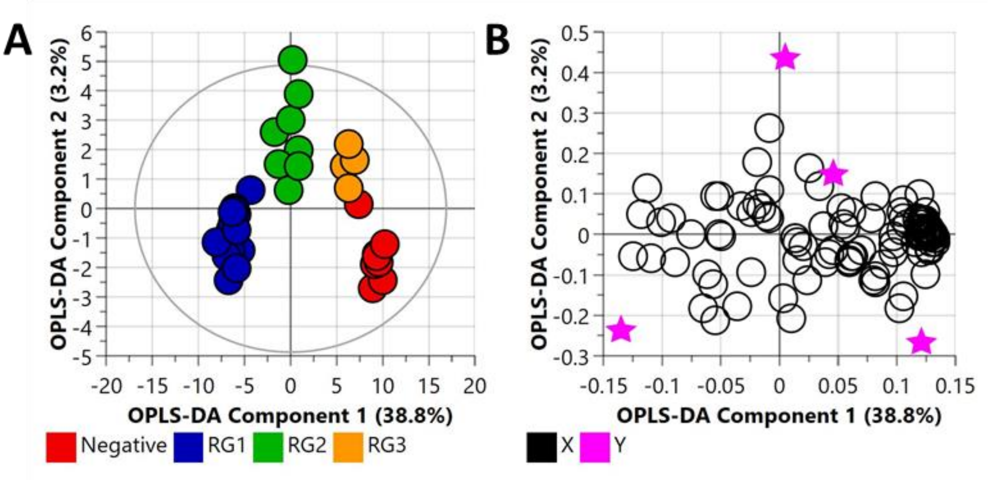

Figure S3. Orthogonal Partial Least Square-Discriminant Analysis (A) scatter plot and (B) loading plot of bacterial pellet samples collected from BPW cultures (n=38; note, the negative group includes *Salmonella*). R^2X (cum) = 0.697, R^2Y (cum) = 0.637, Q^2 = 0.345. Note, the eclipse presented in Figure 2A represents the Hotelling's confidence limit (95%). ***Note:*** *The colored circles in panel "A" represent each analysed samples, while the purple-colored stars in panel "B" indicate the average group position for each sample cluster, with the white circles representing the distribution of metabolite features between these groups.*

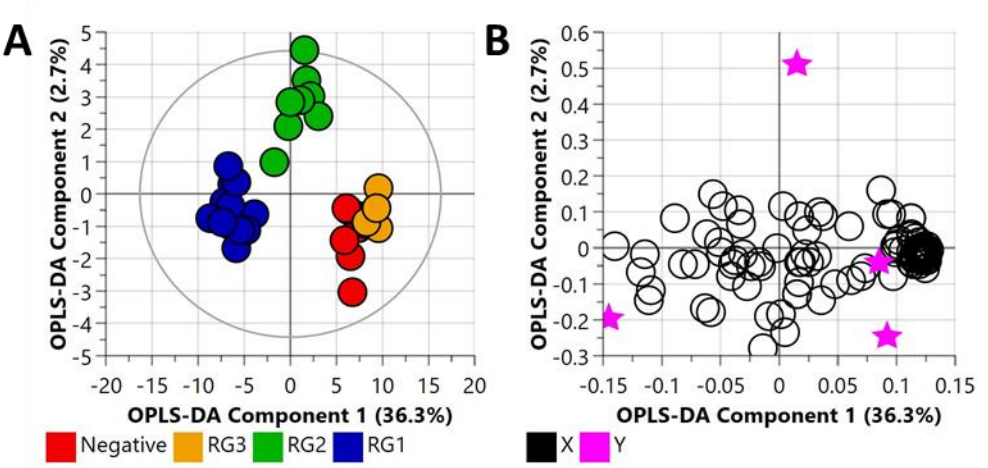

Figure S4. Orthogonal Partial Least Square-Discriminant Analysis (A) scatter plot and (B) loading plot of bacterial supernatant samples collected from BPW cultures (n=38; note, the negative group includes *Salmonella*). R^2X (cum) = 0.765, R^2Y (cum) = 0.845, Q^2 = 0.421. Note, the eclipse presented in Figure 3A represents the Hotelling's confidence limit (95%). ***Note:*** *The colored circles in panel "A" represent each analysed samples, while the purple-colored stars in panel "B" indicate the average group position for each sample cluster, with the white circles representing the distribution of metabolite features between these groups.*

Table S1: Cross validation (CV)-ANOVA of the OPLS-DA bacterial pellet model (Figure S3).

OPLS-DA (Figure S3)	SS	DF	MS	F-test	P value	SD
**Total corr.**	105	105	1			1
**Regression**	46.6142	30	1.55381	1.99595	0.00844	1.24652
**Residual**	58.3858	75	0.778478			0.882314

In this table, SS: sum-of-squares, DF: degrees of freedom, MS: mean squares, SD: standard deviation

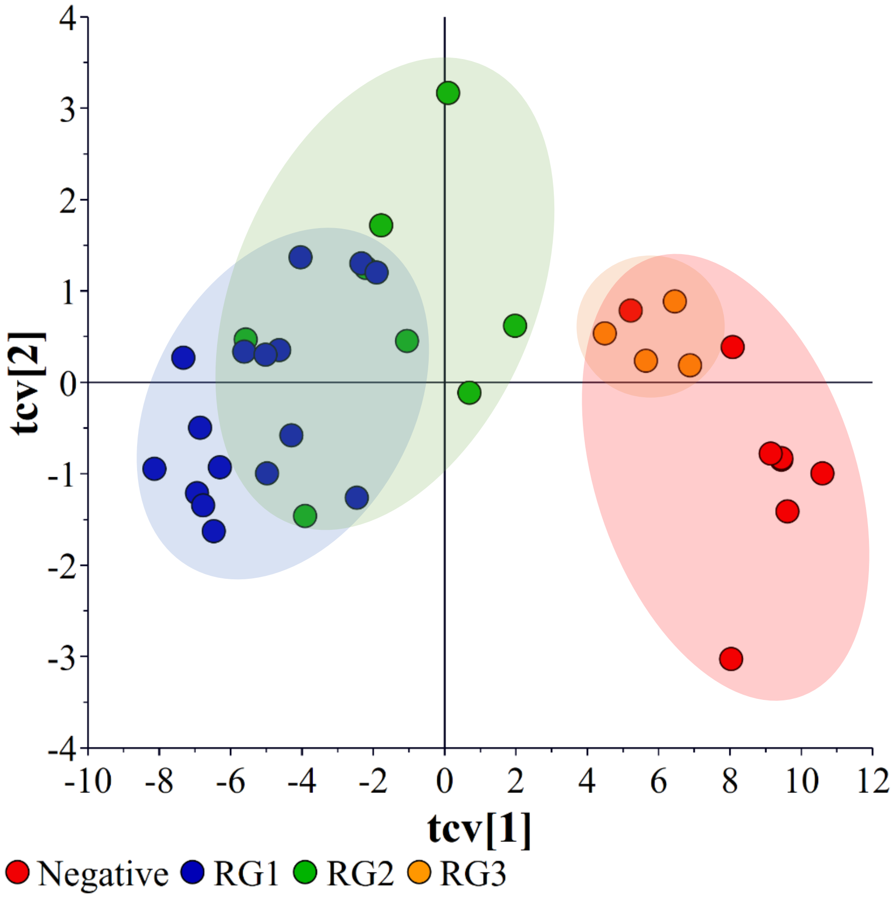

Figure S5: Cross Validation (CV) Scores plots of the OPLS-DA bacterial pellet model. Note, the scatter plot of the cross-validated score vectors is analogous to the scatter plot of regular score vectors (Figure 1) but illustrates the sample stability of each point in relation to the other groups.

Table S2: Cross validation (CV)-ANOVA of the OPLS-DA bacterial supernatant model (Figure S4).

OPLS-DA (Figure S4)	SS	DF	MS	F-test	P value	SD
**Total corr.**	105	105	1			1
**Regression**	36.9623	39	0.947751	0.919366	0.605361	0.973525
**Residual**	68.0377	66	1.03087			1.01532

In this table, SS: sum-of-squares, DF: degrees of freedom, MS: mean squares, SD: standard deviation

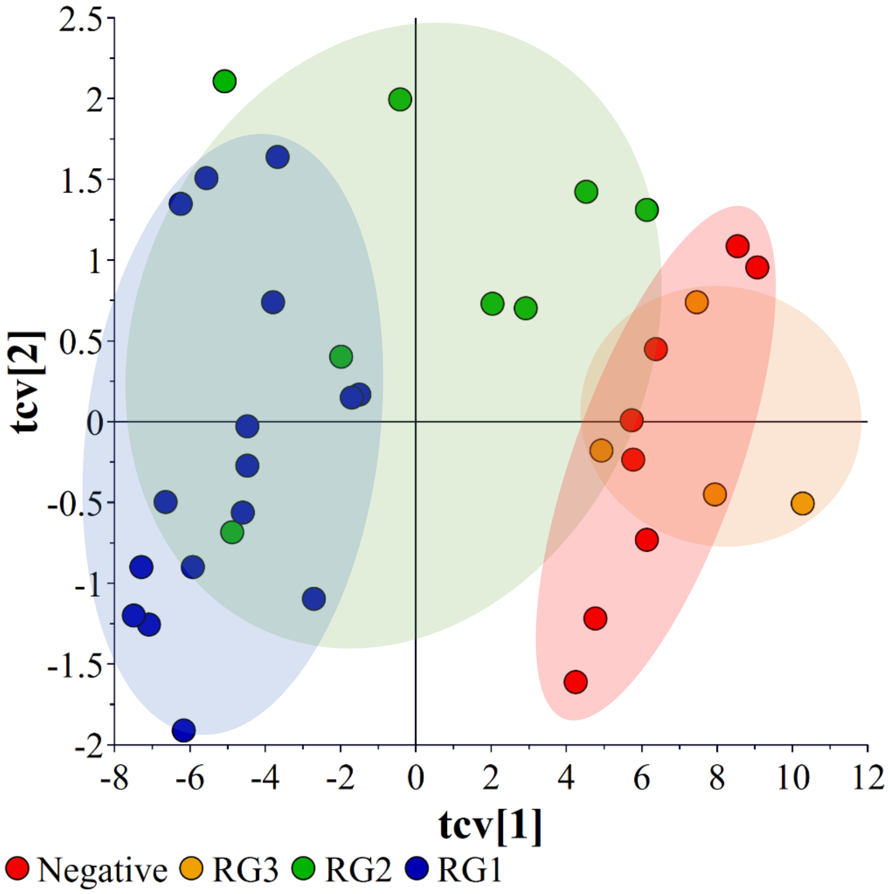

Figure S6: Cross Validation (CV) Scores plots of the OPLS-DA bacterial supernatant model. Note, the scatter plot of the cross-validated score vectors is analogous to the scatter plot of regular score vectors (Figure 1) but illustrates the sample stability of each point in relation to the other groups.

Table S3: Significant metabolites in bacterial pellet samples collected from BPW cultures (n=38; note, the negative group includes *Salmonella*).

Metabolites	Retention Time (min)	Library Score (%)	Fold change	Adjusted P value	Regulation
Compound_18	7.686		36.416	0.000146	up
Compound_14	7.659		35.204	8.28E-06	up
Compound_12	7.61		33.318	0.001508	up
2-amino-2-methyl-1,3-propanediol	8.436	99.6	31.664	9.19E-05	up
D-sphingosine	22.527	83.9	25.018	1.85E-13	up
Compound_73	11.114		24.241	7.12E-10	up
Compound_107	16.617		16.051	0.001213	up
Compound_87	14.312		15.753	0.000257	up
Compound_21	7.873		12.48	5.78E-07	up
Compound_104	16.521		10.114	7.33E-10	up
Behenic acid	23.987	77.6	9.6772	4.92E-05	up
2,3-dihydroxybiphenyl	16.703	97.9	9.5005	0.000127	up
Compound_2	6.741		8.2141	0.004631	up
Compound_96	16.472		8.2125	0.001037	up
Compound_44	9.892		8.1728	3.47E-05	up
Compound_50	9.926		8.1701	7.38E-05	up
Compound_61	10.339		8.0776	0.000731	up
Compound_11	7.598		7.9889	4.92E-05	up
Compound_24	7.952		6.6523	0.000146	up
Compound_38	9.457		6.5173	0.000712	up
Acetohydroxamic acid	7.947	98.5	6.2431	0.000146	up
Compound_27	8.32		6.2068	0.004362	up
3-hydroxyanthranilic acid	17.071	95.1	5.8699	0.000731	up
Compound_51	9.929		5.8517	0.002949	up
Compound_103	16.514		5.5829	1.30E-05	up
Pelargonic acid	10.963	74.4	5.4489	4.92E-05	up
Compound_97	16.48		5.2038	0.007991	up
Compound_9	7.589		5.1091	0.000808	up
Compound_5	7.264		4.5896	0.004927	up
Compound_99	16.495		4.5847	0.000158	up
4-aminophenol	16.501	61.6	4.5141	0.000143	up
Compound_48	9.915		4.4353	0.006312	up
Compound_106	16.591		4.3723	0.000245	up
Compound_49	9.921		4.2725	0.013932	up
DL-2-amino-3-phosphonopropionic acid	16.591	93.8	4.1294	0.000466	up
Compound_16	7.677		3.9466	0.000811	up
Glycolic acid	7.595	76.2	3.9064	0.000182	up
Compound_19	7.757		3.8993	0.001678	up
Halostachine	13.235	83.2	3.8843	0.002475	up
Compound_26	8.022		3.8763	0.000781	up
Compound_82	12.709		3.7364	0.003348	up
Compound_68	10.471		3.5628	0.00204	up
Lauric acid	14.566	71.8	2.2008	0.026559	up

Metabolites	Retention Time (min)	Library Score (%)	Fold change	Adjusted P value	Regulation
2,6-Dihydroxy-4-Methoxytoluene	14.391	99.5	2.0793	0.002067	up
1-hexadecanol	19.099	87.7	2.0793	0.002067	up
Compound_105	16.53		2.0371	0.017356	up
Pipecolic acid	11.159	90.2	0.48959	2.58E-06	down
Trimethyllysine	13.306	74.7	0.41389	1.40E-05	down
Compound_70	10.93		0.37818	3.95E-07	down
L-methionine	13.113	80.5	0.37376	3.95E-07	down
Compound_79	11.783		0.34472	4.07E-07	down
Cytidine	25.107	71.5	0.29306	3.72E-06	down
Compound_111	17.599		0.25147	8.28E-06	down
Compound_76	11.386		0.24706	1.61E-05	down
Compound_77	11.428		0.23977	7.69E-05	down
N-acetyl-ornithine	14.243	92.7	0.23468	2.48E-05	down
Compound_83	12.780		0.21965	1.56E-06	down
Compound_109	16.718		0.21267	1.61E-05	down

Table S4: Significant metabolites in bacterial supernatant samples collected from BPW cultures (n=38; note, the negative group includes *Salmonella*).

Metabolites	Retention Time (min)	Library Score (%)	Fold change	Adjusted P value	Regulation
Compound_103	16.514		38.929	6.63E-05	up
Compound_21	7.873		34.426	3.12E-07	up
Compound_104	16.521		22.265	6.98E-07	up
Compound_107	16.617		19.281	6.74E-07	up
2-amino-2-methyl-1,3-propanediol	8.436	99.6	18.976	0.002331	up
Compound_87	14.312		17.041	0.000872	up
2,3-dihydroxybiphenyl	16.703	97.9	10.598	0.000988	up
Compound_105	16.53		6.9911	0.000729	up
Compound_38	9.457		6.8391	0.013821	up
Compound_44	9.892		6.6591	0.004764	up
Compound_102	16.508		6.6434	0.020342	up
Compound_96	16.472		6.4177	0.00816	up
Compound_48	9.915		6.2733	0.002057	up
behenic acid	23.987	77.6	5.4042	0.001537	up
Compound_18	7.686		5.3986	0.000729	up
2,3-butanediol	6.753	99.0	5.0483	0.001857	up
Compound_4	6.964		5.0482	0.001857	up
Compound_14	7.659		4.9289	0.002907	up
Compound_51	9.929		4.6055	0.002113	up
Compound_49	9.921		4.5877	0.025265	up
pelargonic acid	10.963	74.4	3.8394	0.018413	up
Compound_11	7.598		3.7539	0.002784	up
Compound_61	10.339		3.6594	0.012926	up
Compound_24	7.952		3.4676	0.003368	up
Compound_12	7.61		3.4617	0.034869	up
4-aminophenol	16.501	61.6	3.3531	0.000729	up
acetohydroxamic acid	7.947	98.5	3.3156	0.003907	up
Compound_5	7.264		3.1738	0.012242	up
Compound_9	7.589		3.073	0.005124	up
Compound_99	16.495		2.865	0.001857	up
Compound_106	16.591		2.813	0.00348	up
Compound_26	8.022		2.7512	0.013821	up
glycolic acid	7.595	76.2	2.6654	0.002113	up
3-hydroxyanthranilic acid	17.071	95.1	2.5867	0.031998	up
Compound_27	8.32		2.5864	0.031998	up
Halostachine	13.235	83.2	2.5024	0.025265	up
Compound_19	7.757		2.5024	0.025265	up
Compound_22	7.932		2.4491	0.00816	up
Compound_16	7.677		2.4334	0.031998	up
DL-2-amino-3-phosphonopropionic acid	8.436	99.6	2.4136	0.019143	up
D-sphingosine	22.527	83.9	2.3341	0.001613	up
Compound_68	10.471		2.2822	0.049952	up
Compound_73	11.114		2.2638	0.003307	up

Metabolites	Retention Time (min)	Library Score (%)	Fold change	Adjusted P value	Regulation
Compound_76	11.386		0.48847	0.007747	down
Compound_77	11.428		0.48806	0.018413	down
Compound_111	17.599		0.46467	0.004764	down
epsilon-caprolactam	8.099	92.2	0.31819	0.003841	down
Compound_109	16.718		0.30493	0.001613	down
N-acetyl-ornithine	14.243	92.7	0.25191	0.000804	down

Table S5: ANOVA analysis of bacterial pellet samples collected from BPW cultures.

Metabolites	Library Score (%)	Pubchem ID	KEGG ID	F-statistic	P value	FDR	Fisher's LSD
D-sphingosine	83.9	5280335	C00319	55.978	1.05E-15	1.12E-13	RG1 > Negative; RG2 > Negative; RG1 > RG2; RG1 > RG3; RG2 > RG3
L-methionine	80.5	6137	C00073	15.094	4.74E-07	8.38E-06	Negative > RG1; Negative > RG2; RG3 > RG1; RG3 > RG2
Pipecolic acid	90.2	439227	C00408	12.302	4.31E-06	4.24E-05	Negative > RG1; Negative > RG2; RG3 > RG1; RG3 > RG2
Cytidine	71.5	596	NA	12.163	4.84E-06	4.27E-05	Negative > RG1; Negative > RG2; RG3 > RG1; RG3 > RG2
N-acetyl-ornithine	92.7	439232	C00437	10.016	3.08E-05	2.17E-04	Negative > RG1; Negative > RG2; RG3 > RG1; RG3 > RG2
Trimethyllysine	74.7	159660	NA	9.5429	4.72E-05	2.94E-04	Negative > RG1; Negative > RG2; RG3 > RG1; RG3 > RG2
Behenic acid	77.6	8215	C08281	8.806	9.31E-05	4.93E-04	RG1 > Negative; RG2 > Negative; RG1 > RG3; RG2 > RG3
Conduritol epoxide	50.9	119054	NA	8.3638	1.41E-04	6.80E-04	Negative > RG1; Negative > RG2; RG3 > RG1
Pelargonic acid	74.4	8158	C01601	8.0606	1.89E-04	8.42E-04	RG1 > Negative; RG2 > Negative; RG1 > RG3
2-amino-2-methyl-1,3-propanediol	99.6	1531	C11260	7.4813	3.31E-04	1.35E-03	RG1 > Negative; RG2 > Negative; RG1 > RG3
2,6-Dihydroxy-4-Methoxytoluene	99.5	238769	NA	7.2436	4.19E-04	1.48E-03	RG1 > Negative; Negative > RG3; RG1 > RG3; RG2 > RG3
1-hexadecanol	87.7	2682	C00823	7.2436	4.19E-04	1.48E-03	RG1 > Negative; Negative > RG3; RG1 > RG3; RG2 > RG3
2,3-dihydroxybiphenyl	97.9	254	C02526	6.817	6.41E-04	2.12E-03	RG1 > Negative; RG2 > Negative; RG1 > RG3; RG2 > RG3
4-aminophenol	61.6	403	C02372	6.5666	8.25E-04	2.65E-03	RG1 > Negative; RG2 > Negative; RG1 > RG3; RG2 > RG3
Acetohydroxamic acid	98.5	1990	C06808	6.4834	8.98E-04	2.72E-03	RG1 > Negative; RG2 > Negative; RG1 > RG3; RG2 > RG3
Glycolic acid	76.2	757	C00160	6.2912	1.09E-03	3.13E-03	RG1 > Negative; RG2 > Negative; RG1 > RG3; RG2 > RG3
DL-2-amino-3-phosphonopropionic acid	93.8	3857	C05672	5.3854	2.82E-03	7.11E-03	RG1 > Negative; RG2 > Negative; RG1 > RG3; RG2 > RG3
Glycine	91.3	750	C00037	5.2306	3.32E-03	8.19E-03	Negative > RG1; Negative > RG2; RG3 > RG1
3-hydroxyanthranilic acid	95.1	86	C00632	5.0685	3.95E-03	9.31E-03	RG1 > Negative; RG2 > Negative; RG1 > RG3
Acetol	87.7	8299	C05235	5.0174	4.18E-03	9.62E-03	Negative > RG1; RG3 > RG1
DL-3-aminoisobutyric acid	99.9	64956	C05145	3.9524	1.34E-02	2.59E-02	RG1 > Negative; RG1 > RG3
Lauric acid	71.8	3893	C02679	3.7978	1.60E-02	2.95E-02	RG1 > RG2; RG1 > RG3
Halostachine	83.2	913	C03711	3.7885	1.62E-02	2.95E-02	RG1 > Negative; RG2 > Negative; RG1 > RG3; RG2 > RG3
L-proline	85.1	145742	C00148	3.4894	2.26E-02	3.69E-02	Negative > RG1
Acetoacetate	65.7	96	C00164	3.2799	2.87E-02	4.51E-02	RG1 > RG2; RG1 > RG3

Table S6: ANOVA analysis of bacterial supernatant samples collected from BPW cultures.

Metabolites	Library Score (%)	Pubchem ID	KEGG ID	F statistic	P value	FDR	Fisher's LSD
D-sphingosine	83.9	5280335	C00319	26.235	3.42E-10	1.81E-08	RG1 > Negative; Negative > RG3; RG1 > RG2; RG1 > RG3; RG2 > RG3
pelargonic acid	74.4	8158	C01601	9.2378	6.24E-05	8.26E-04	RG1 > Negative; RG1 > RG2; RG1 > RG3
2,3-dihydroxybiphenyl	97.9	254	C02526	8.6094	1.12E-04	1.32E-03	RG1 > Negative; RG1 > RG2; RG1 > RG3
DL-3-aminoisobutyric acid	99.9	64956	C05145	8.2684	1.55E-04	1.64E-03	RG1 > Negative; RG1 > RG2; RG1 > RG3
4-aminophenol	61.6	403	C02372	7.4483	3.42E-04	3.02E-03	RG1 > Negative; RG1 > RG2; RG1 > RG3
behenic acid	77.6	8215	C08281	6.8098	6.45E-04	4.56E-03	RG1 > Negative; RG1 > RG2; RG1 > RG3
N-acetyl-ornithine	92.7	439232	C00437	6.0757	1.37E-03	8.85E-03	Negative > RG1; Negative > RG2; RG3 > RG1; RG3 > RG2
acetoacetate	65.7	96	C00164	6.0289	1.43E-03	8.85E-03	RG3 > Negative; RG3 > RG1; RG3 > RG2
2,3-butanediol	99	262	NA	5.5697	2.32E-03	1.12E-02	RG1 > Negative; RG2 > Negative; RG1 > RG3; RG2 > RG3
L-alanine	79.1	5950	C00041	5.5275	2.42E-03	1.12E-02	RG1 > Negative; RG1 > RG3
glycolic acid	76.2	757	C00160	5.1918	3.46E-03	1.47E-02	RG1 > Negative; RG1 > RG3; RG2 > RG3
2-amino-2-methyl-1,3-propanediol	99.6	1531	C11260	4.9224	4.63E-03	1.75E-02	RG1 > Negative; RG1 > RG3
acetohydroxamic acid	98.5	1990	C06808	4.377	8.39E-03	2.74E-02	RG1 > Negative; RG1 > RG3; RG2 > RG3
epsilon-caprolactam	92.2	7768	C06593	4.2255	9.92E-03	3.09E-02	Negative > RG1; RG3 > RG1; RG3 > RG2

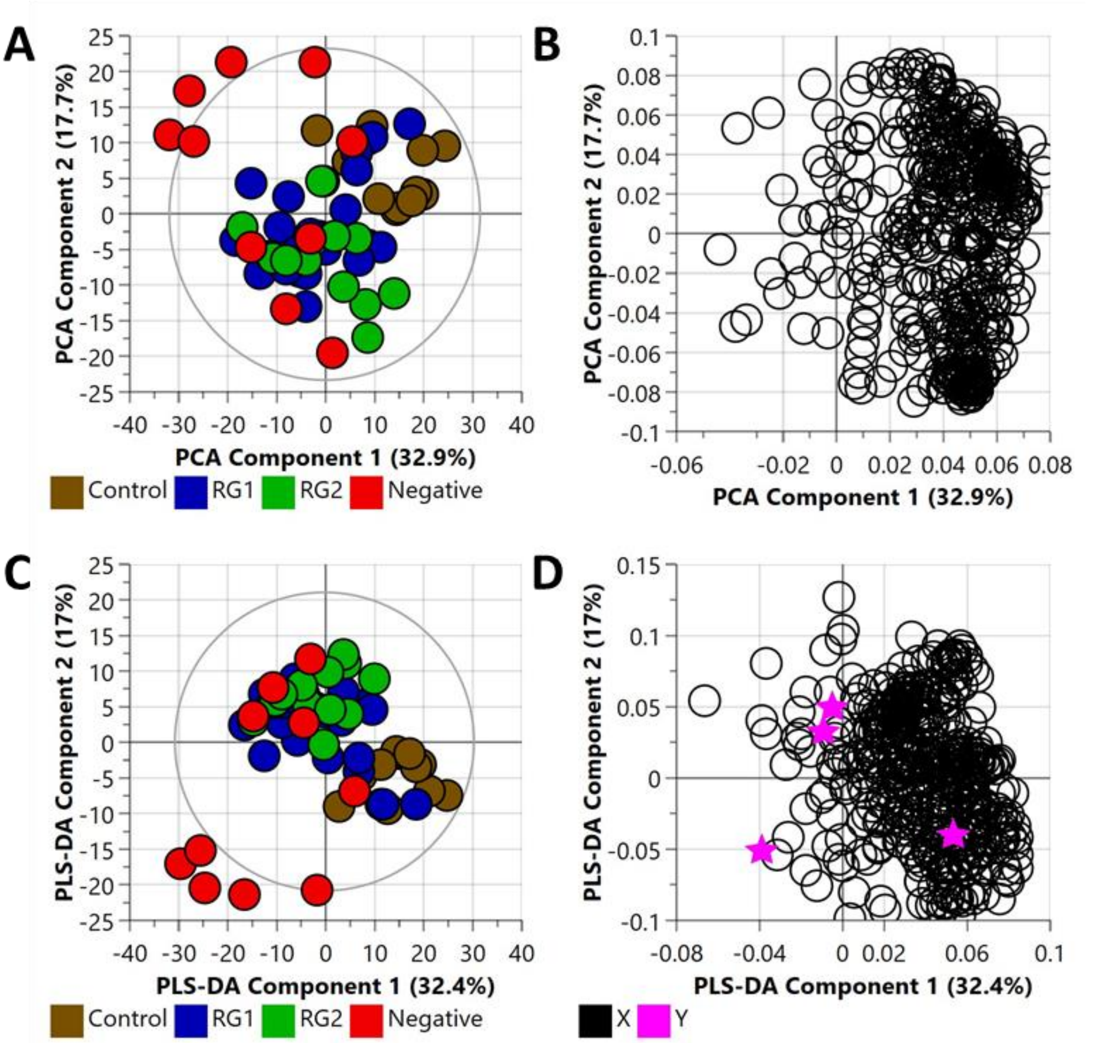

Figure S7. Principal component analysis (PCA) (R2X (cum) = 0.714, Q2 = 0.572) and partial least square-discriminant analysis (PLS-DA) (R2X (cum) = 0.671, R2Y (cum) = 0.757, Q2 = 0.431) of bacterial pellet samples collected from inoculated spinach samples. *Note: the eclipse presented in Figure S4A and S4C represents the Hotelling's confidence limit (95%). The colored circles in panel "A" and "C" represent each analyzed samples, while the purple-colored stars in panel "B" and "D" indicate the average group position for each sample cluster, with the white circles representing the distribution of metabolite features between these groups.*

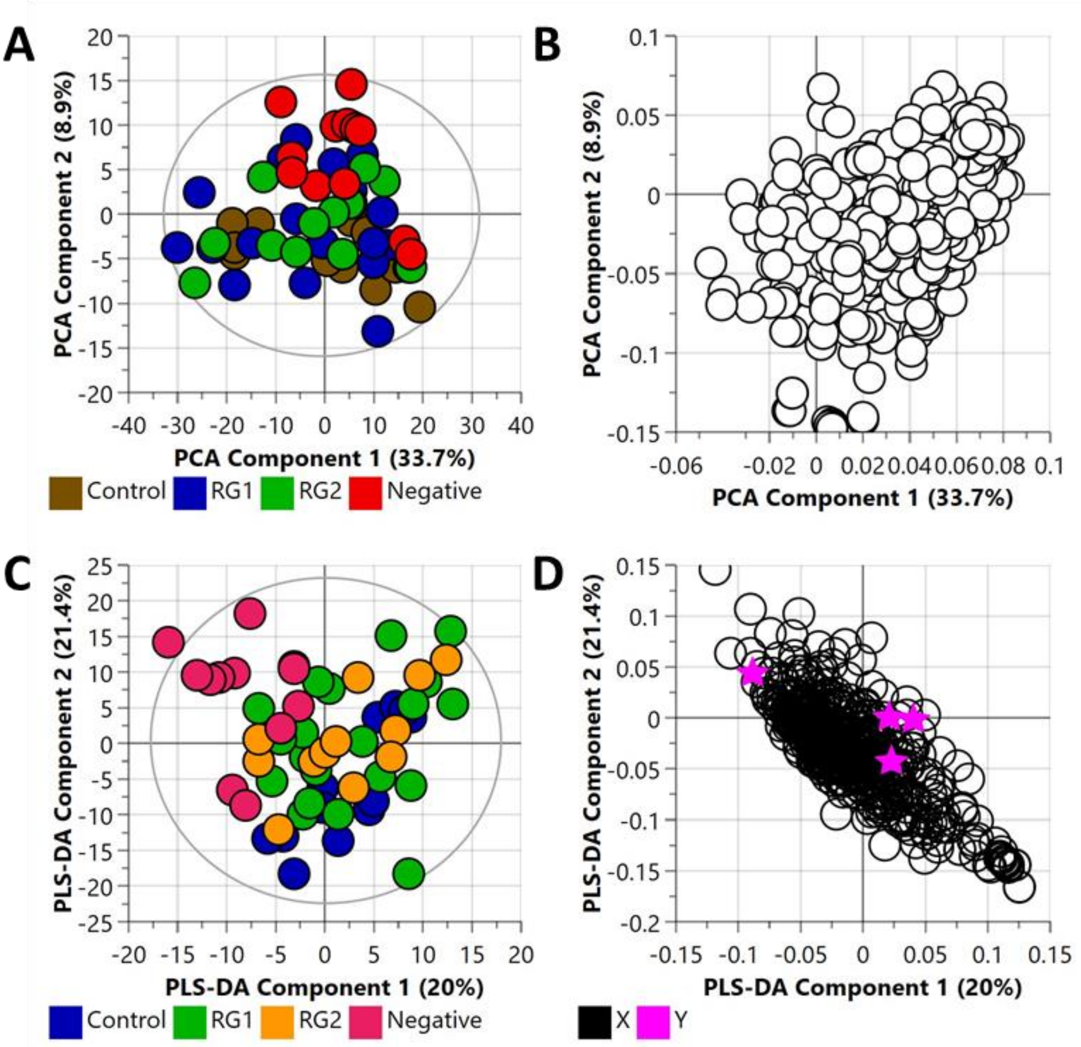

Figure S8. Principal component analysis (PCA) (R2X (cum) = 0.712, Q2 = 0.464) and partial least square-discriminant analysis (PLS-DA) (R2X (cum) = 0.52, R2Y (cum) = 0.746, Q2 = 0.329) of bacterial supernatant samples collected from inoculated spinach samples. *Note: the eclipse presented in Figure S5A and S5C represents the Hotelling's confidence limit (95%). The colored circles in panel "A" and "C" represent each analysed samples, while the purple-colored stars in panel "B" and "D" indicate the average group position for each sample cluster, with the white circles representing the distribution of metabolite features between these groups.*

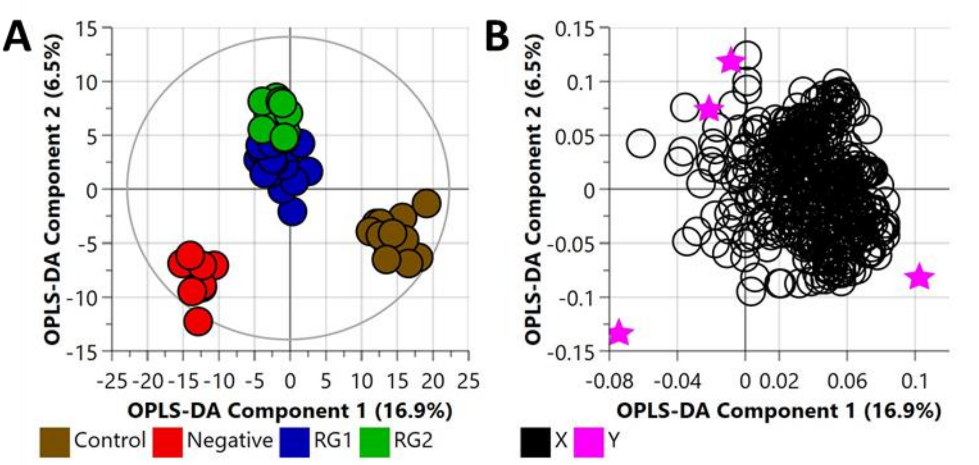

Figure S9. Orthogonal Partial Least Square-Discriminant Analysis (A) scatter plot and (B) loading plot of bacterial pellet samples collected from inoculated spinach samples (n=58). R^2X (cum) = 0.763, R^2Y (cum) = 0.920, Q^2 = 0.314. Note, the eclipse presented in Figure 4A represents the Hotelling's confidence limit (95%). ***Note:*** *The colored circles in panel "A" represent each analysed samples, while the purple-colored stars in panel "B" indicate the average group position for each sample cluster, with the white circles representing the distribution of metabolite features between these groups.*

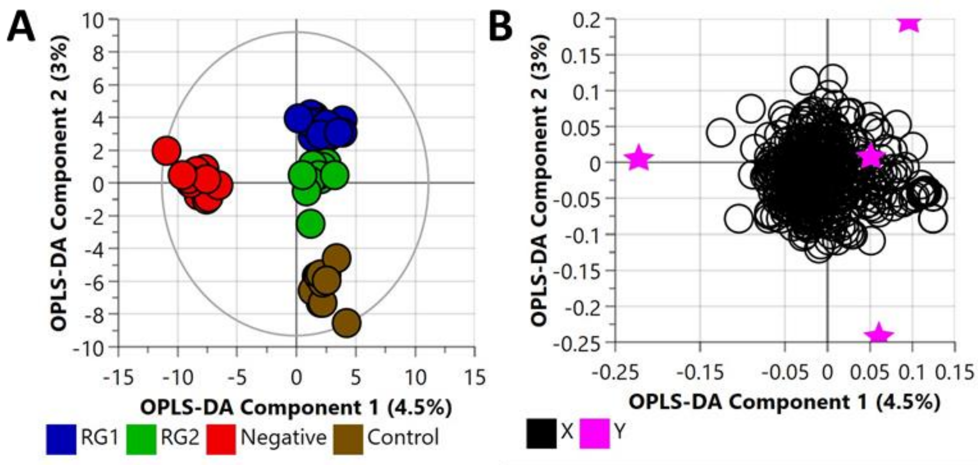

Figure S10. Orthogonal Partial Least Square-Discriminant Analysis (A) scatter plot and (B) loading plot of bacterial supernatant samples collected from inoculated spinach samples (n=58). R^2X (cum) = 0.635, R^2Y (cum) = 0.945, Q^2 = 0.429. Note, the eclipse presented in Figure 5A represents the Hotelling's confidence limit (95%). ***Note:*** *The colored circles in panel "A" represent each analysed samples, while the purple-colored stars in panel "B" indicate the average group position for each sample cluster, with the white circles representing the distribution of metabolite features between these groups.*

Table S7: Cross validation (CV)-ANOVA of the OPLS-DA inoculated spinach pellet model.

OPLS-DA (Inoculated spinach pellet model)	SS	DF	MS	F-test	P value	SD
**Total corr.**	171	171	1			1
**Regression**	79.5159	54	1.47252	1.88322	0.00234138	1.21347
**Residual**	91.4841	117	0.781916			0.88426

In this table, SS: sum-of-squares, DF: degrees of freedom, MS: mean squares, SD: standard deviation

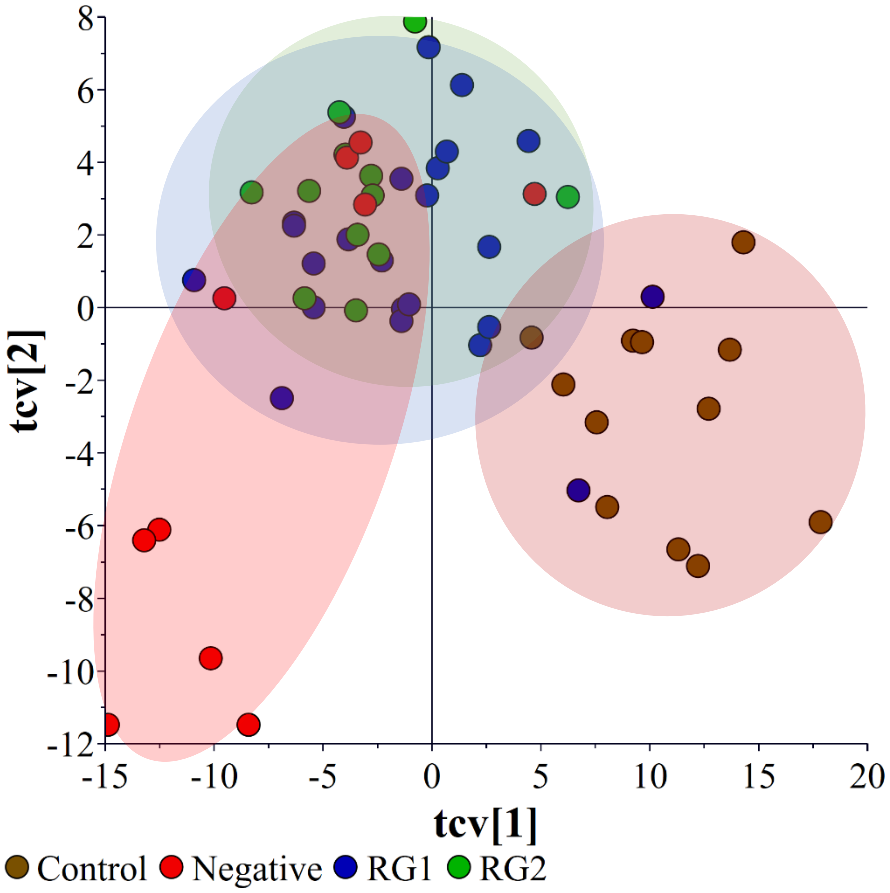

Figure S11: Cross Validation (CV) Scores plots of the OPLS-DA bacterial supernatant model. Note, the scatter plot of the cross-validated score vectors is analogous to the scatter plot of regular score vectors (Figure 1) but illustrates the sample stability of each point in relation to the other groups.

Table S8: Cross validation (CV)-ANOVA of the OPLS-DA inoculated spinach supernatant model.

OPLS-DA (Inoculated spinach supernatant model)	SS	DF	MS	F-test	P value	SD
**Total corr.**	165	165	1			1
**Regression**	71.263	54	1.31969	1.56272	0.0246481	1.14878
**Residual**	93.737	111	0.844478			0.918955

In this table, SS: sum-of-squares, DF: degrees of freedom, MS: mean squares, SD: standard deviation

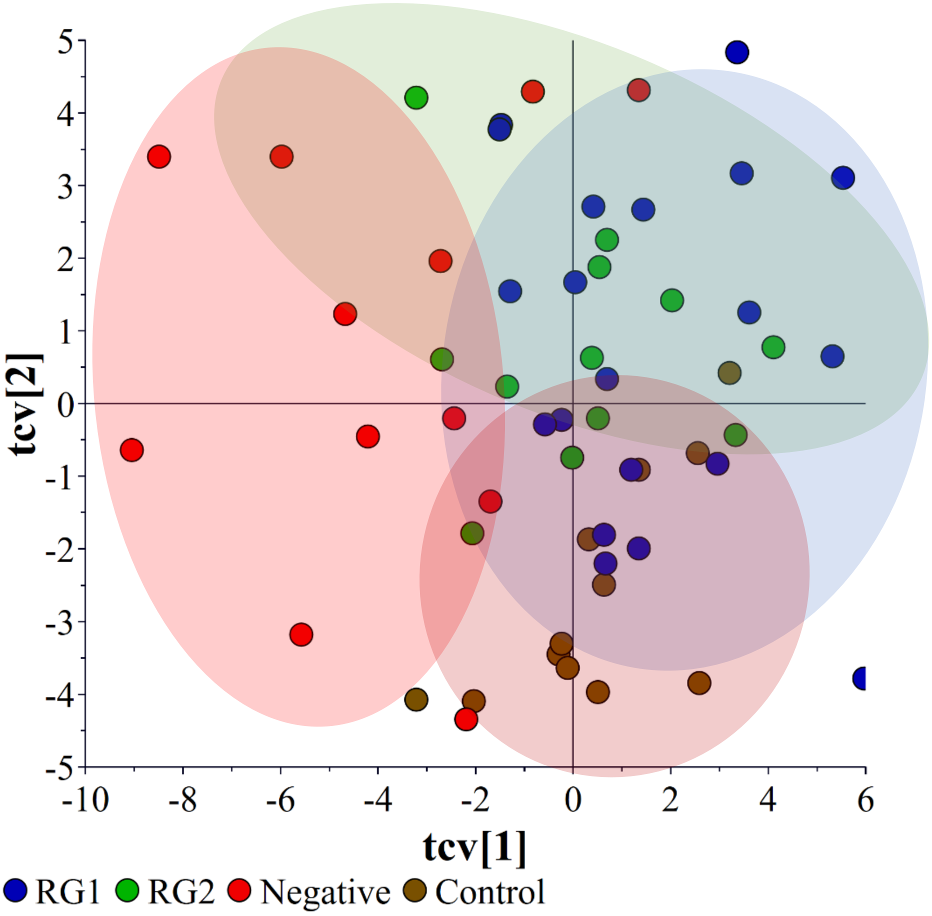

Figure S12: Cross Validation (CV) Scores plots of the OPLS-DA inoculated spinach supernatant model. Note, the scatter plot of the cross-validated score vectors is analogous to the scatter plot of regular score vectors (Figure 1) but illustrates the sample stability of each point in relation to the other groups.

Table S9: Significant metabolites identified in bacterial pellet from RG1-inoculated spinach samples.

Metabolites	Library Score (%)	Human Metabolome Database	KEGG	P value	Fold change	Regulation
L-methionine	86.7	HMDB0000696	C00073	0.0045	2.36097	Up
4-hydroxycinnamic acid	87.8	HMDB0002035	C00811	0.01295	2.09518	Up
Putrescine	99.4	HMDB0001414	C00134	0.005317	0.490402	Down
Citric acid	66.1	HMDB0000094	C00158	0.012117	0.487528	Down
L-threonine	91.8	HMDB0000167	C00188	0.001047	0.483517	Down
Inosine	95.5	HMDB0000195	C00294	0.024845	0.46021	Down
O-phosphocolamine	96.2	HMDB0000224	C00346	0.049315	0.452313	Down
Phosphoric acid	99.1	HMDB0002142	C00009	0.011027	0.451882	Down
Stearic acid	96.5	HMDB0000827	C01530	0.000723	0.451536	Down
L-glutamic acid	99.2	HMDB0000148	C00025	0.000483	0.447883	Down
Methyl Caprate	66.9	HMDB0033848	No result	0.002917	0.446061	Down
Myristic acid	95.7	HMDB0000806	C06424	0.000247	0.4452	Down
Linoleic acid	94.8	HMDB0000673	C01595	0.001348	0.439841	Down
Methyl-beta-D-galactopyranoside	90.7	No result	C03619	0.000918	0.432168	Down
Behenic acid	93.0	HMDB0000944	C08281	0.004614	0.409336	Down
Spermidine	93.4	HMDB0001257	C00315	0.001029	0.409256	Down
Acetol	92.9	HMDB0006961	C05235	0.037705	0.402971	Down
Glycerol 1-phosphate	99.5	No result	C03189	0.000989	0.390908	Down
Methyl linolenate	86.9	No result	No result	0.000984	0.388655	Down
3-phosphoglyceric acid	86.5	HMDB0060180	C00197	0.049638	0.386142	Down
L-proline	97.5	HMDB0000162	C00148	0.001112	0.339837	Down
Guanosine	94.2	HMDB0000133	C00387	0.018204	0.307033	Down
Hypoxanthine	64.4	HMDB0000157	C00262	0.000188	0.303996	Down
Adenosine	97.1	HMDB0000050	C00212	0.004923	0.29823	Down
Eicosane	95.8	HMDB0059909	No result	0.005486	0.280899	Down
Methyl Palmitate	87.2	HMDB0061859	C16995	0.000534	0.271417	Down

Metabolites	Library Score (%)	Human Metabolome Database	KEGG	P value	Fold change	Regulation
3,7-Dihydroxyflavone	88.2	HMDB0134547	No result	0.000689	0.266666	Down
Aspartic acid	95.0	HMDB0000191	C00049	0.000139	0.262345	Down
Nitrazepam	92.0	HMDB0015534	C07487	0.000594	0.254608	Down
Phytol	90.3	No result	No result	0.000145	0.254329	Down
Adenine	87.7	HMDB0000034	C00147	4.50E-05	0.252255	Down
Cytosine	87.2	HMDB0000630	C00380	0.009951	0.247596	Down
Citraconic acid	99.3	HMDB0000749	C01732	9.96E-06	0.243214	Down
5,6-dihydro-5-methyluracil	88.8	HMDB0000079	C00906	0.000315	0.24311	Down
N-methylalanine	97.7	HMDB0094692	C02721	8.90E-06	0.237281	Down
Epsilon-caprolactam	54.3	HMDB0062769	C06593	1.54E-06	0.231002	Down
1-hexadecanol	83.8	HMDB0003424	C00823	0.000528	0.230051	Down
L-ornithine	95.0	HMDB0000214	C00077	0.000239	0.230008	Down
Lauric acid	92.0	HMDB0000638	C02679	0.000154	0.226546	Down
L-valine	94.5	HMDB0000883	C00183	8.20E-08	0.226404	Down
Cellotetraose	99.6	No result	C02013	1.23E-06	0.213537	Down
L-lysine	97.7	HMDB0000182	C00047	6.19E-06	0.213351	Down
Methyl palmitoleate	76.1	No result	No result	0.024343	0.20616	Down
L-norleucine	98.0	HMDB0001645	C01933	6.13E-08	0.171248	Down
Tyrosine	95.4	HMDB0000158	C00082	6.40E-08	0.161078	Down
Xanthine	88.4	HMDB0000292	C00385	4.85E-07	0.152953	Down
L-tryptophan	93.4	HMDB0000929	C00078	4.51E-07	0.090689	Down

Table S10: Significant metabolites identified in bacterial pellet from RG2-inoculated spinach samples.

Metabolites	Library Score (%)	Human Metabolome Database	KEGG	P value	Fold change	Regulation
4-hydroxyphenylacetic acid	89.3	HMDB0000020	C00642	0.00289	0.250798	Down
5-aminovaleric acid	96.1	HMDB0003355	C00431	5.33E-05	0.326284	Down
Adenine	87.7	HMDB0000034	C00147	1.49E-06	0.141199	Down
Citric acid	66.1	HMDB0000094	C00158	0.000386	0.269741	Down
1,3-diaminopropane	84.9	HMDB0000002	C00986	0.000867	0.352664	Down
Cytosine	87.2	HMDB0000630	C00380	0.01771	0.122146	Down
D-Ala-D-Ala2	97.1	No result	No result	2.89E-05	0.396973	Down
Glycerol 1-phosphate	99.5	No result	C03189	3.64E-05	0.279513	Down
Hypoxanthine	64.4	HMDB0000157	C00262	0.007248	0.340268	Down
Phenethylamine	92.4	HMDB0012275	C05332	0.018288	0.483771	Down
Phosphoric acid	99.1	HMDB0002142	C00009	0.009625	0.411665	Down
Putrescine	99.4	HMDB0001414	C00134	1.24E-05	0.347272	Down
Spermidine	93.4	HMDB0001257	C00315	0.004649	0.418087	Down
Xanthine	88.4	HMDB0000292	C00385	1.43E-06	0.093152	Down
1-hexadecanol	83.8	HMDB0003424	C00823	0.002572	0.157649	Down
Lauric acid	92.0	HMDB0000638	C02679	0.000927	0.122289	Down
Thymidine	72.1	HMDB0000273	C00214	0.033617	0.168474	Down
Trans-4-hydroxy-L-proline	86.4	HMDB0000725	C01157	0.041933	0.231242	Down
L-serine	97.5	HMDB0000187	C00065	0.003752	0.423077	Down
Aspartic acid	95.0	HMDB0000191	C00049	2.58E-05	0.140077	Down
L-lysine	97.7	HMDB0000182	C00047	3.27E-06	0.139814	Down
Inosine	95.5	HMDB0000195	C00294	0.001703	0.155599	Down
Tyrosine	95.4	HMDB0000158	C00082	2.29E-07	0.127933	Down
L-methionine	86.7	HMDB0000696	C00073	0.000106	3.08852	Up
L-ornithine	95.0	HMDB0000214	C00077	0.001884	0.117089	Down
L-valine	94.5	HMDB0000883	C00183	0.00029	0.34979	Down

Metabolites	Library Score (%)	Human Metabolome Database	KEGG	P value	Fold change	Regulation
L-threonine	91.8	HMDB0000167	C00188	3.12E-03	0.372341	Down
L-tryptophan	93.4	HMDB0000929	C00078	4.92E-05	0.062864	Down
Guanosine	94.2	HMDB0000133	C00387	0.000264	0.061949	Down
2-hydroxybiphenyl	93.2	HMDB0032582	C02499	0.001607	0.193868	Down
Epsilon-caprolactam	54.3	HMDB0062769	C06593	2.43E-06	0.176559	Down
Methyl Palmitate	87.2	HMDB0061859	C16995	0.000227	0.130236	Down
Behenic acid	93.0	HMDB0000944	C08281	0.000872	0.258145	Down
Eicosane	95.8	HMDB0059909	No result	0.0025	0.046264	Down
Acetol	92.9	HMDB0006961	C05235	0.02656	0.194565	Down
Dioctyl phthalate	90.0	No result	C03690	3.28E-05	0.434437	Down
Hexanoic acid	98.5	HMDB0000535	C01585	0.002573	0.288155	Down
Methyl linolenate	86.9	No result	No result	0.000702	0.289092	Down
4-(2-hydroxyethyl)phenol	90.4	HMDB0004284	C06044	6.63E-14	0.13254	Down
Myristic acid	95.7	HMDB0000806	C06424	0.000162	0.313572	Down
Phendimetrazine	62.8	HMDB0015519	No result	0.000406	0.238234	Down
L-norleucine	98.0	HMDB0001645	C01933	2.32E-07	0.124477	Down
L-glutamic acid	99.2	HMDB0000148	C00025	4.98E-06	0.331559	Down
Adenosine	97.1	HMDB0000050	C00212	0.003319	0.14406	Down
5,6-dihydro-5-methyluracil	88.8	HMDB0000079	C00906	0.000585	0.206062	Down
Methyl-beta-D-galactopyranoside	90.7	No result	C03619	0.003075	0.342085	Down
3-Hydroxy-3',4'-Dimethoxyflavone	82.8	No result	No result	0.028674	7.18358	Up
L-proline	97.5	HMDB0000162	C00148	4.40E-05	0.17678	Down
3-phosphoglyceric acid	86.5	HMDB0060180	C00197	0.016312	0.262672	Down
Cellotetraose	99.6	No result	C02013	3.89E-05	0.130264	Down
4-hydroxycinnamic acid	87.8	HMDB0002035	C00811	0.000889	2.54912	Up
Citraconic acid	99.3	HMDB0000749	C01732	4.81E-06	0.212431	Down
D-sphingosine	68.8	No result	C00319	0.002491	0.340146	Down
Squalene	72.4	No result	No result	0.016476	0.413256	Down

Metabolites	Library Score (%)	Human Metabolome Database	KEGG	P value	Fold change	Regulation
Linoleic acid	94.8	HMDB0000673	C01595	0.002483	0.36246	Down
N-methylalanine	97.7	HMDB0094692	C02721	0.000153	0.190999	Down
3,7-Dihydroxyflavone	88.2	HMDB0134547	No result	5.52E-05	0.032102	Down
Phytol	90.3	No result	No result	0.000137	0.111386	Down

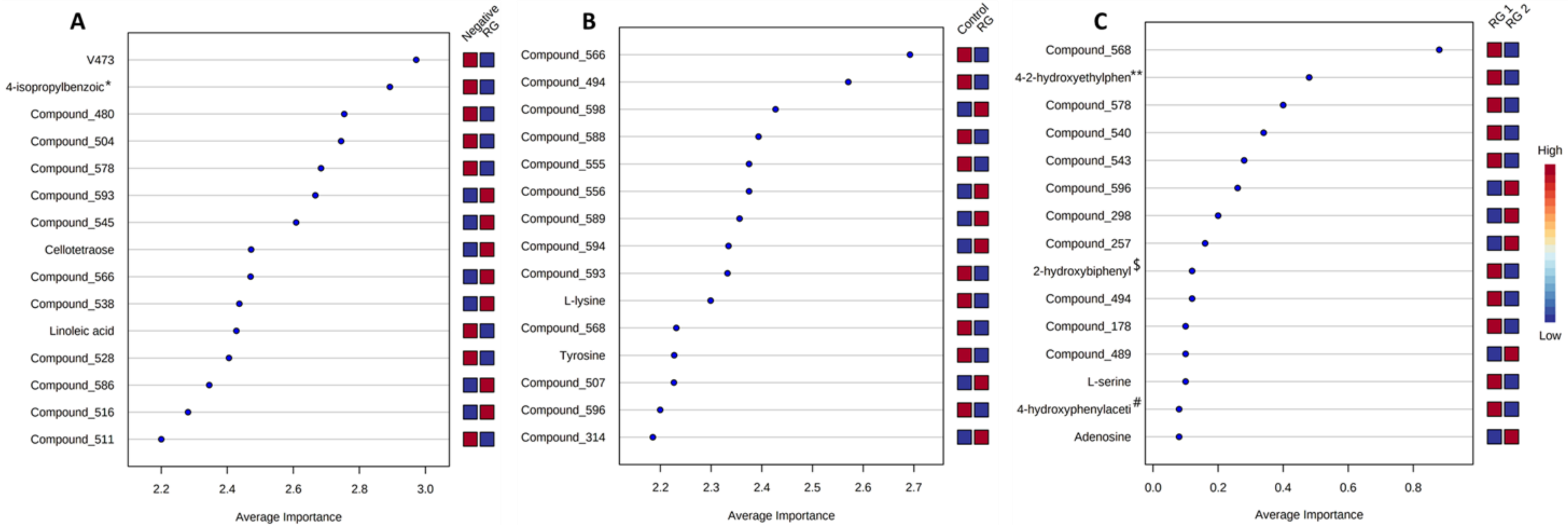

Figure S13. The plots indicate the top 15 validated biomarkers, as analysed by PLS-DA classification and feature ranking through a Monte-Carlo cross-validation (MCVV) method. The comparisons consisted of (A) Negative vs RG and (B) Control vs RG and, (C) RG 1 vs RG 2. ***Note****: The symbols denote* *4-isopropyl benzoic acid, **4-(2-hydroxyethyl)phenol, $2-hydroxybiphenyl and #4-hydroxyphenylacetic acid (A). **Note**: metabolites annotated "Compound_###" are indicative of unknown compounds.*
